# One Year of Coronavirus Disease 2019 (COVID-19) in Brazil: A Political and Social Overview

**DOI:** 10.5334/aogh.3182

**Published:** 2021-05-18

**Authors:** Matheus Negri Boschiero, Camila Vantini Capasso Palamim, Manoela Marques Ortega, Renan Marrichi Mauch, Fernando Augusto Lima Marson

**Affiliations:** 1 Laboratory of Cell and Molecular Tumor Biology and Bioactive Compounds, So Francisco University, Bragana Paulista, SP, Brazil; 2 Laboratory of Human and Medical Genetics, So Francisco University, Bragana Paulista, SP, Brazil; 3 Laboratory of Translational Medicine, Center for Investigation in Pediatrics, School of Medical Sciences, University of Campinas. Campinas, SP, Brazil

## Abstract

**Background:**

Coronavirus Disease 2019 (COVID-19) became the deadliest pandemic of the new millennium. One year after it became a pandemic, the current COVID-19 situation in Brazil is an example of how the impacts of a pandemic are beyond health outcomes and how health, social, and political actions are intertwined.

**Objectives:**

We aimed to provide an overview of the first year of the COVID-19 pandemic in Brazil, from a social and political point of view, and to discuss the perspectives from now on.

**Methods:**

This is a narrative review using official, scientific (PubMed, Medline, and SciELO databases) and publicly available data. Press articles were also used that contain important information not found in these databases.

**Findings:**

We address the impacts of COVID-19 in different regions of Brazil, on indigenous populations, health care workers, and how internal social contrasts impacted the pandemics advance across the country. We also discuss key points that culminated in the countrys failed management of the COVID-19 spread, such as poor management of the public health care system, disparities between public and private health care infrastructure, lack of mass testing and viral spread tracking, lack of preparedness and planning to implement strict isolation and social distancing measures, and, most importantly, political instability, a deteriorating Health Ministry and sabotaging attitudes of the countrys president, including anti-scientific actions, underplaying COVID-19 severity, spreading and powering fake news about the pandemic, promoting knowingly inefficient medications for COVID-19 treatment, and interference in collective health policies, including the countrys vaccination plan.

**Conclusions:**

After one year of COVID-19 and a disastrous management of the disease, Brazil has more than 11 million cases, 270,000 deaths, and the highest number of daily deaths due to COVID-19 in the world, most of which could have been avoided and can be credited to negligence of municipal, state, and federal authorities, especially President Jair Messias Bolsonaro. Unfortunately, the country is an example of what not to do in a pandemic setting.

**Key Points:**

One year after COVID-19 was declared a pandemic, Brazil had the second higher number of cases and deaths, and the highest number of daily deaths due to the disease.
Lack of massive testing, non-stringent and ineffective collective health policies, poor management of the public health care system, and political instability were the main drivers of the countrys flawed management of the COVID-19 advancement.
Anti-science and sabotaging actions by government had a pivotal role in the countrys current situation.
Brazil has a large territory and is marked by social contrasts among different regions and states, which showed contrasting data regarding the impact caused by COVID-19.
COVID-19 databases and data sharing are important to provide an overview of epidemiological aspects of the disease; however, Brazil lacks standardization in these datasets.

## 1. Introduction

Coronavirus disease 2019 (COVID-19), the viral infection caused by the new coronavirus strain severe acute respiratory syndrome (SARS)-Coronavirus-2 (SARS-CoV-2) was discovered in the end of 2019 in the city of Wuhan, China, and, in a matter of months, became the deadliest pandemic of the new millennium [1]. Although most COVID-19 cases are asymptomatic infection or mild-to-moderate respiratory disease [2,3], the virus fast transmission in the community led to a striking impact in public health worldwide, which was mainly characterized by overload in intensive care units (ICUs) and collapse of health care systems [4]. This called for the implementation of social isolation and safety measures, including partial lockdowns in many countries, which, in turn, have caused dramatic changes in peoples lifestyles and several economic consequences. The fast viral spread and its devastating consequences also led the scientific community to speed up the research on disease treatment and vaccines in a way never before seen in history. Vaccine trials skipped several conventional stages and bureaucratic steps were dropped in order to release the emergence use of newly developed vaccines in Europe, North America, and Asia [5].

To date, one year after the World Health Organization (WHO) declared COVID-19 as a pandemic, vaccines are available and many countries have started vaccinating their populations, and treatment alternatives for severe COVID-19 have also been developed that mainly consist of immunosuppressant agents targeting the intense inflammatory reaction following infection [6,7]. However, as vaccination should take long to reach most of the worlds population, social distancing, isolation, lockdowns and safety measures such as using facial masks and quarantine are still the best way to prevent the viral spread. As for health authorities worldwide, having the population respect the implemented measures, setting up the logistics for COVID-19 testing and vaccination, and working in conjunction with other sectors to prevent social gatherings in public spaces remain as major challenges, especially in an era of quick dissemination of contents, including the ones from unreliable sources, which is facilitated by internet and social network applications. Developing diagnostic tests that can be massively applied to allow better screening for infection is also a challenge, since the gold-standard test for COVID-19 diagnosis, SARS-CoV-2 reverse transcriptase polymerase chain reaction (RT-PCR), can only be performed in specialized laboratories and, therefore, is not accessible for the population in many countries.

The situation in Brazil is a good scenario to understand how the impacts caused by a pandemic of this magnitude go beyond health outcomes. The countrys socio-economic inequalities, political instability, concentrated population and structurally defective public health system steeply contribute to maximize the pandemic effects on peoples lives, the countrys finances and the countrys reputation abroad. In the present article, we aimed to provide an overview of the first year of the COVID-19 pandemic, in Brazil, from a social and political point of view, and to discuss the perspectives from now on.

## 2. Methods

This is a narrative review. Our article *per se* is scientific in nature, and we based it mainly on official data and scientific literature, using the Worldometer, Our World in Data, PubMed, Medline, and SciELO databases. However, it is undeniable that the attitudes and conduct of political leaders around the globe were decisive for the pandemics course in different countries, shaping the behavior of their populations towards it and the way it was faced, which also has direct impact on science as a whole. In this sense, there were many controversies in Brazil, and we consider that the role of the Brazilian press and of the international press, reporting the way in which the Brazilian government has dealt with the pandemic, bringing to the attention of the lay population the causes and consequences of governmental actions, has been crucial. For this reason, we have also referred to articles from newspapers and websites aimed at the non-scientific public.

## 3. Perspective

### 3.1. Overview of the COVID-19 cases worldwide and in Brazil

On March 12, 2021, exactly one year after being declared as a pandemic, there were 118,058,503 confirmed cases of COVID-19 worldwide, 258,735 of which were reported in the last 24 hours, and 2,621,046 confirmed deaths (2.22% lethality rate), 10,018 of which happened in the last 24 hours [8]. However, COVID-19 does not affect the world homogenously. The American continent has the highest number of cases (52,386,995 cases) and deaths (1,258,134, 2.40% lethality rate), whereas the Western Pacific region has the lowest figures, with 1,694,716 cases and 30,076 deaths (1.77% lethality rate) [8] (***Table 1***). Despite having the highest death toll, the lethality rate in the American continent is similar to what has been shown worldwide (2.40% vs. 2.22%) [8].

**Table 1:** Total of confirmed cases and deaths due to COVID-19 in each region and globally (adapted from WHO, 2021) [8].


REGION	CONFIRMED CASES	DEATHS (% LETHALITY RATE)

Africa	2,924,244	74,143 (2.54)

Americas	52,386,995	1,258,134 (2.40)

Eastern Mediterranean	6,793,641	149,400 (2.20)

Europe	40,438,291	897,540 (2.22)

South-East Asia	13,819,871	211,740 (1.53)

Western Pacific	1,694,716	30,076 (1.77)

**Globally**	118,058,503	2,621,046 (2.22)


WHO, World Health Organization; %, percentage.

**Figure 1:**
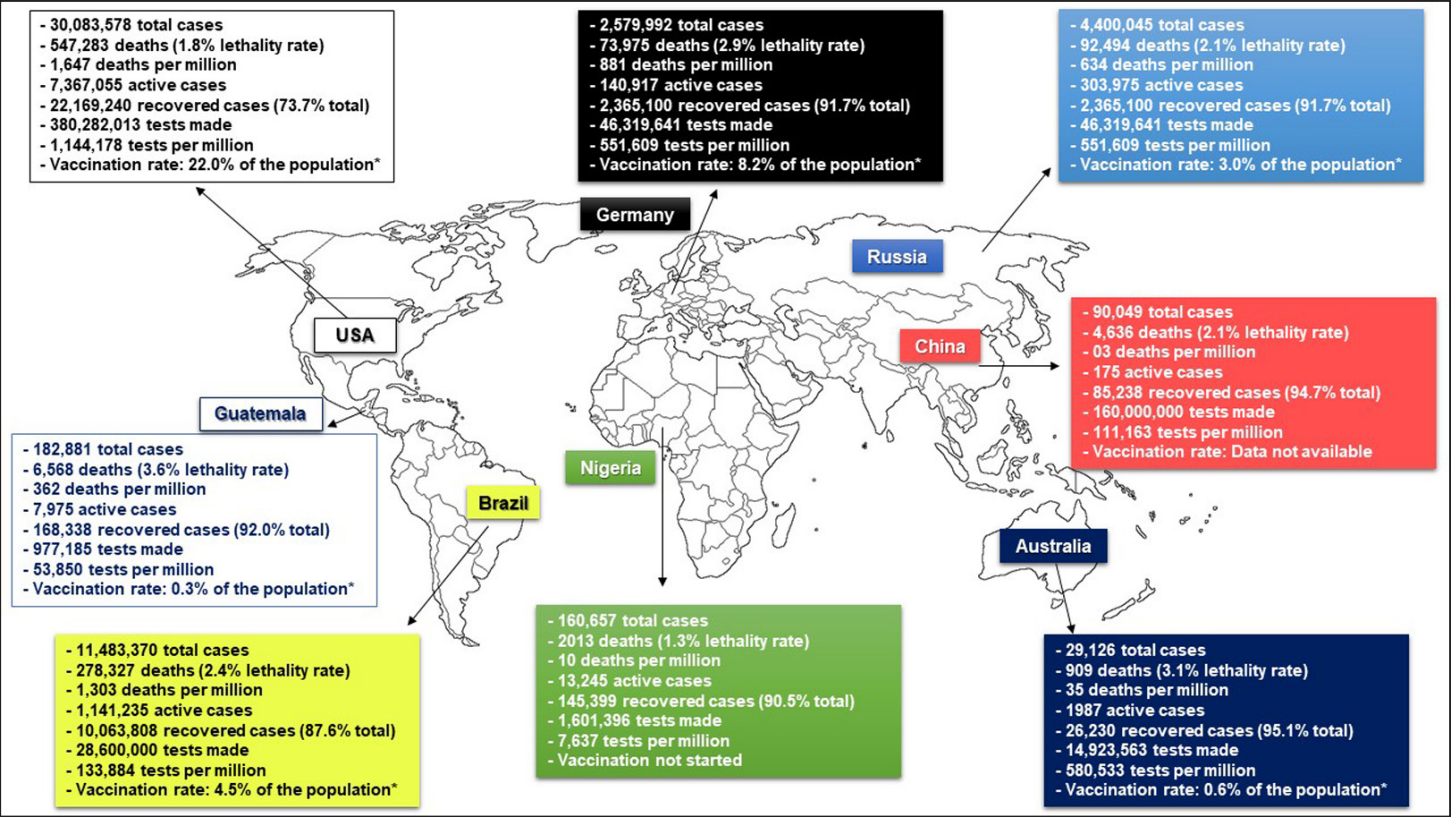
Comparison of COVID-19 data between Brazil and the most populated country of each continent (North America, Central America, Europe, Africa, Asia, Oceania) and Russia, as for March 12 2021. Total number of COVID-19 patients, total number of deaths (and total death rate), number of and deaths per one million of inhabitants, total number of active COVID-19 cases, total number of recovered cases (and total recovery rate), total number of SARS-CoV-2 RT-PCR tests made in the country, and number of inhabitants of SARS-CoV-2 RT-PCR tests made per one million inhabitants and vaccination rate. *People who have received at least one shot of a vaccine. The data were retrieved from WorldOMeter (*https://www.worldometers.info/coronavirus/country/*) and Our World In Data (*http://ourworldindata.org/policy-responses-covid*) [10,24].

Brazil had 11,277,717 COVID-19 confirmed cases (ranking third worldwide), with 75,412 in the last 24 hours, and 272,899 confirmed deaths (ranking second worldwide), 2,283 of which happened in the last 24 hours [9]. On March 12, 2021, Brazil reached more than 2,000 daily deaths, the highest number in one year. In addition, Brazil had 1,052,579 active cases (ranking third worldwide), 10,231,690 total recovered cases (ranking third worldwide), 52,618 cases per one million inhabitants (ranking 39th worldwide), and 1,279 deaths per one million inhabitants (ranking 25th worldwide). Brazil performed SARS-CoV-2 RT-PCR tests for 28,600,000 samples (ranking 12th worldwide), which corresponds to only 133,892 per one million inhabitants (ranking 121th worldwide) [10]. Compared with the most populated countries in each continent, Brazil generally has more deaths and fewer tests per one million inhabitants (***Figure 1***), indicating not only that Brazil is one of the most affected countries, but also that the number of cases is underreported. In fact, a study using a serological approach estimated that the number of infected people is about six times higher than the number notified to the Ministry of Health [11], which means that the real number of COVID-19 cases in Brazil can be around 67 million, which, in turn, raises concern about the number of unreported deaths. Also, preliminary data from the Oswaldo Cruz Foundation (Fiocruz) showed that the prevalence of acute respiratory distress syndrome (ARDS) in Brazil increased from 0.8/100,000 inhabitants in 2019 to 7.5/100,000 inhabitants in 2020, which is likely to be explained by several COVID-19 cases being diagnosed as ARDS from unknown source, corroborating the underreporting hypothesis [12].

Brazil and the United States of America (USA) are the most affected countries in the Americas. Together, the two countries account for 40,142,442 confirmed COVID-19 cases (76.63% of the cases in the Americas) and 796,151 deaths (63.28% of the deaths in the Americas) [8,10]. There are differences between Brazil and USA in the management of the COVID-19 outbreak. One of these differences concerns the number of SARS-CoV-2 RT-PCR assays, which is visibly higher in the USA [10]. However, after President Joe Biden took office as the new president of the USA on January 2021, several measures have been taken in order to proper face this pandemic, such as rejoin the WHO, a safe, effective, and equitable vaccination campaign, and expanding the use of masks, tests, and treatment. Even though these actions were made in January, it is clear the number of deaths is already decreasing in the USA [13]. Interestingly, a lower number of tests and poor governmental health policies have shown to be associated with a higher mortality due to COVID-19, which may explain the higher death rate in Brazil [14].

**Figure 2:**
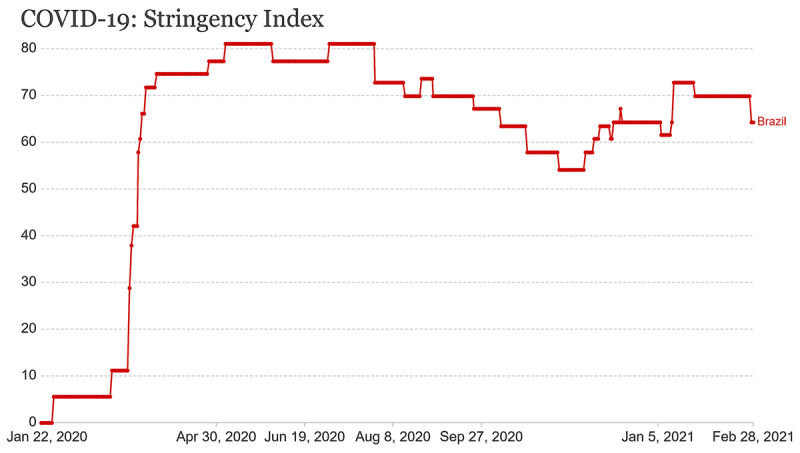
COVID-19: Stringency Index from Brazil. The graph represents a composite measure based on nine response indicators including school closures, workplaces closures, and travels ban, rescaled to value from 0 to 100 (100 = strictest). If policies vary at the subnational level, the index is shown as the response level of the strictest sub-region. Source: Hale, Webster, Petherick, Phillips, and Kira (2020). Oxford COVID-19 Government Response Tracker Last Update 15 March 2021, 08:00 (London time). Note: The index records the number and strictness of government policies and should be interpreted as scoring the appropriateness or effectiveness of a countrys response. Data search: OurWorldInData/coronavirus [24].

**Figure 3:**
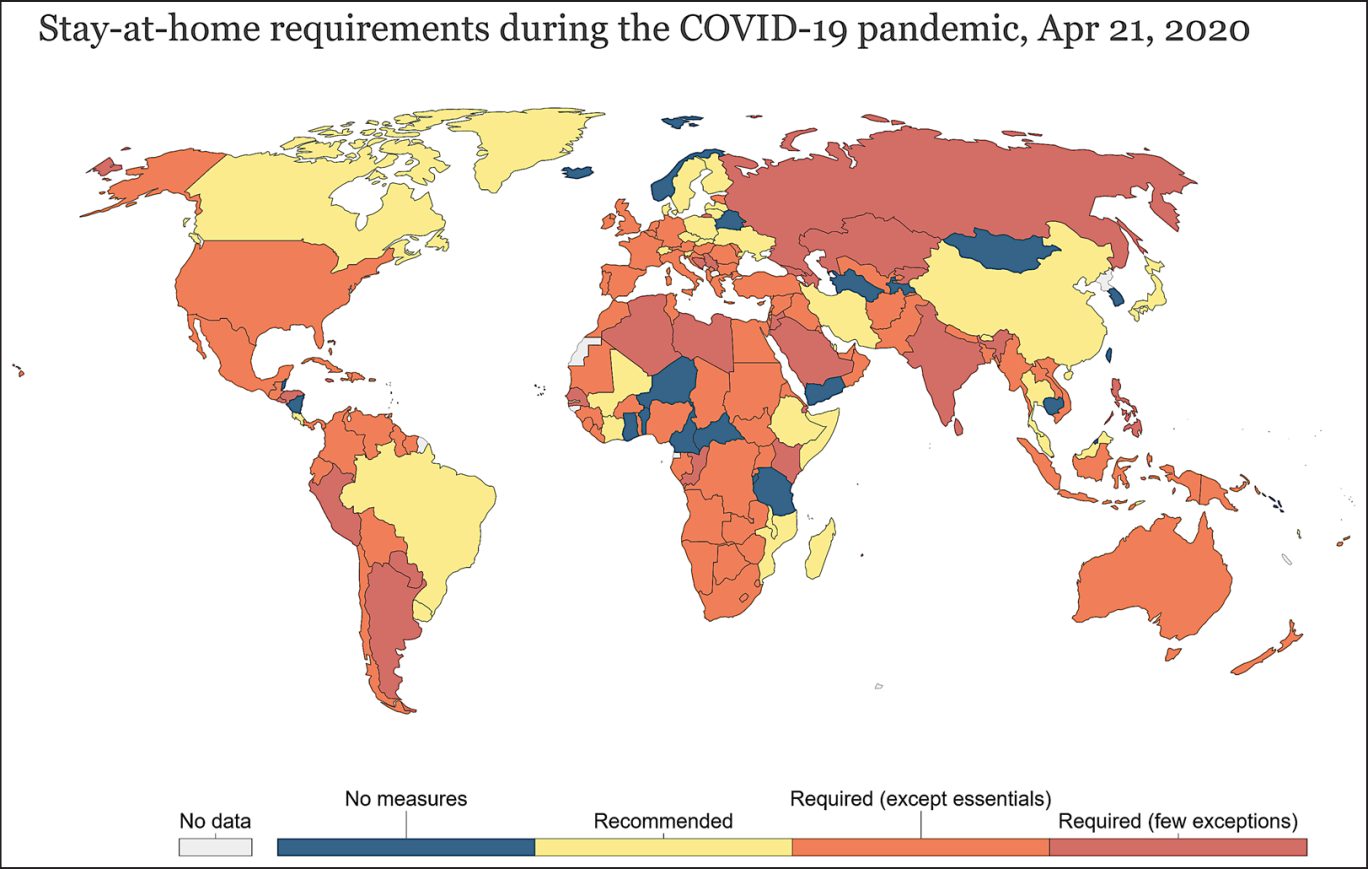
Stay-at-Home requirements during the COVID-19 pandemic. Source: Hale, Webster, Petherick, Phillips, and Kira (2020). Oxford COVID-19 Government Response Tracker Last Update 15 March 2021, 08:00 (London time). Note: There may be sub-national or regional differences in restrictions. The policy categories show may not apply at all sub-national levels. A country is coded as having these restrictions if a least some sub-national regions have implemented them. Data search: OurWorldInData/coronavirus [24].

Also, compared to the most populous countries [15,16,17,18,19,20,21,22], the measures taken by the Brazilian government were not efficient to contain the advancement of COVID-19, and took longer to enter into force (***Figure 2***). After the first case, on December 31, 2019, COVID-19 was rapidly reported by the Chinese government, on January 31, 2020, to the WHO, which, in turn, warned about the risk of a new pandemic. The first case in Brazil was identified on February 26, 2020 [9], when thousands of cases had already been reported across Europe [8]. In the first COVID-19 wave, health care units in Brazil started to collapse by mid-April, more than one month after the pandemic was declared, and, by then, circulation restrictions were not mandatory yet (***Figures 2*** and ***3***) [23,24]. This means that, from the moment when COVID-19 was reported to the WHO, Brazil had one and a half months to implement containment measures and at least two months to prepare for the worst scenario.

### 3.2. COVID-19 cases by States, Federal District and Regions in Brazil

Brazil has a large geographic area that comprises 26 states and the Federal District, divided into five major regions (North, Northeast, Midwest, Southeast, and South). These regions are contrasting in terms of population number and density, number of COVID-19 cases, ICU facilities, ICUs per number of inhabitants, and infrastructure to conduct high numbers of the SARS-CoV-2 RT-PCR tests [9,25]. Concomitantly, Brazil has many social contrasts regarding access to basic sanitation, health care, transport, education, and security, which reflects the contrasting human development indexes among different states and regions, and even among different spots within the same state or city. All these factors play a role in the risk of COVID-19 infection and dissemination, as well as in the ability of a given population to follow the isolation and social distance measures [26,27,28,29,30,31]. According to official reports [9], on March 12, 2021, the Southeast region had the highest number of confirmed COVID-19 cases. Among the federation states, So Paulo, which has both the largest population and highest population density, accounted for the highest number of reported cases, while Rio de Janeiro had the highest lethality rate [9,32]. The number of cases per 100,000 inhabitants also varies across Brazil. The countrys average number was 5,366.6 per 100,000 inhabitants for COVID-19. The State of Roraima had the highest number, while the State of Maranho had the lowest. Among the five regions, the highest and lowest numbers were reported in the Midwest and Southeast Regions, respectively. The average number of deaths was 129.9 per 100,000 inhabitants, the highest one being reported in the State of Amazonas and the lowest one in the State of Maranho. Among the five regions, the highest number of deaths per 100,000 inhabitants was reported in the North Region (157.2 deaths per 100,000 inhabitants) and the lowest in the Northeast Region (***Table 2***). The State of Amazonas was the first one to have its health care system collapsed in the first Covid-19 wave [33].

**Table 2:** Demographic characteristics of COVID-19 in Brazil on March 12, 2021 [9,32].


STATES AND THE FEDERAL DISTRICT	CASES	DEATHS	CASE FATALITY RATE	CASES/100,00 INHABITANTS	DEATHS/100,00 INHABITANTS	ADHESION FOR SOCIAL ISOLATION (%)	RESPIRATORY VENTILATORS DISTRIBUTED BY MINISTRY OF HEALTH	RESPIRATORY VENTILATORS BILLED BY STATES	TOTAL AMOUNT PAID (R$) PER MILLION	AMOUNT PAID (R$) PER UNIT MILLION ON (THOUSAND)	TOTAL RT-PCR TESTS (TOTAL PAID R$)	TOTAL QUICK TESTS

**Brasil**	11,277,717	272,889	2.42	5,366.6	129.9						14,725,497 (748,194,799)	8,836,305

**Midwest region**	1,196,427	24,014	2.01	7,341.4	147.4						972,888 (49,661,242)	770,120

Gois	425,206	9,332	2.19	6,058.5	133.0	36.6	413				156,272 (8,281,255)	252,240

Mato Grosso	266,939	6,097	2.28	7,660.8	175.0	38.8	216	120(50 national)	7.4 (2.2)	61.7 (44)	203,808 (9,972,691)	116,540

Distrito Federal	312,956	5,048	1.61	10,379.0	167.4	40.3	250				274,680 (14,013,668)	300,640

Mato Grosso do Sul	191,326	3,537	1.85	6,884.7	127.3	37.1	155	11(+ 25 portable)	1.5 (1.4)	135 (55.7)	338,128 (17,393,626)	100,700

**South region**	2,183,813	35,899	1.64	7,285.2	119.8						2,887,556 (147,765,115)	1,162,040

Santa Catarina	717,454	8,377	1.17	10,013.6	116.9	38.7	98	50*	33*	165*	334,264 (18,230,532)	266,140

Rio Grande do Sul	720,461	14,363	1.99	6,332.5	126.2	43.6	486				571,284 (27,305,593)	468,300

Paran	745,898	13,159	1.76	6,523.5	115.1	37.6	544				1,982,008 (102,228,989)	427,600

**North region**	1,226,601	28,982	2.36	6,655.1	157.2						1,440,140 (73,190,252)	606,000

Acre	61,394	1,094	1.78	6,961.3	124.0	43	170				129,724 (6,666,490)	26,560

Rondnia	162,818	3,278	2.01	9,161.4	184.4	41	248				208,696 (10,412,872)	58,060

Tocantins	122,426	1,623	1.33	7,783.6	103.2	35	115				168,196 (8,423,864)	59,200

Amazonas	328,763	11,431	3.48	7,932.3	275.8	40.6	222	28	2.9	103.5	237,668 (11,761,688)	162,060

Amap	87,095	1,169	1.34	10,298.2	138.2	41.8	125				325,516 (15,139,839)	23,840

Par	379,196	9,171	2.42	4,407.8	106.6	38.2	409	400	50.4	126	260,236 (14,809,776)	258,940

Roraima	84,909	1,216	1.43	14,016.9	200.7	39.4	162	NI	NI	215.4	110,104 (5,975,729)	17,340

**Northeast region**	2,617,780	60,158	2.30	4,586.8	105.4						3,718,896 (187,054,809)	2,103,440

Alagoas	138,065	3,150	2.28	4,137.0	94.4	41.7	185				106,884 (5,947,398)	112,920

Pernambuco	313,227	11,269	3.60	3,277.4	117.9	44.5	205	500	NI	NI	314,552(17,009,411)	335,640

Bahia	730,542	12,961	1.77	4,911.8	87.1	42.2	491	300	48.7	162.4	836,932(40,490,720)	531,300

Paraba	234,254	4,832	2.06	5,829.9	120.3	41.8	285	30	4.9	164	155,548 (8,147,262)	164,260

Sergipe	157.340	3,072	1.95	6,844.8	133.6	42.5	140				571,728 (26,433,375)	79,760

Piau	182,650	3,545	1.94	5,580.1	108.3	43.1	105				204,492(10,183,466)	147,780

Cear	456,948	12,087	2.65	5,003.8	132.4	42.1	268				1,039,460 (54,432,045)	318,600

Maranho	226,172	5,413	2.39	3,196.7	76.5	39.1	281				215,412 (10,812,174)	233,800

Rio Grande do Norte	178,582	3,829	2.14	5,092.4	109.2	40.4	274	NI	NI	~70.4	273,888 (13,598,954)	179,380

**Southeast region**	4,053,096	123,836	3.06	4,586.4	140.1						5,705,712(290,523,379)	3,816,545

So Paulo	2,164,066	63,010	2.91	4,712.8	137.2	38.5	838	3,000^b^	550	189.2	2,536,944 (133,193,867)	1,743,880

Esprito Santo	340,808	6,656	1.95	8,480.7	165.6	38.6	210				178,728 (8,856,083)	202,300

Rio de Janeiro	601,666	34,083	5.66	3,484.9	197.4	40.8	993	1,000*	183.5*	183.5*	2,228,728(114,858,287)	1,049,245

Minas Gerais	946,556	20,087	2.12	4,471.5	94.9	37.8	561	1,047	51	48.7	761,312 (33,615,140)	821,120


NI, not informed. The data was collected at *https://covid.saude.gov.br*. Accessed on March 12, 2021. The number for social isolation was obtained at InLoco [56]. The number of respiratory ventilators distributed by Ministry of Health was collected on *https://www.gov.br/pt-br/noticias/saude-e-vigilancia-sanitaria/2020/07/governo-federal-ja-entregou-mais-de-8-4-mil-ventiladores-pulmonares* [9]. *, the purchase was canceled due to possible irregularities; **, the information was retrieved during July 2020. ^a^, 1,820 were purchased with a cost of R$ 242,200,000.

### 3.3. Indigenous people and COVID-19 in Brazil: Demographic data

There are 896,917 indigenous (native Brazilian descent) individuals of 305 different ethnic groups (with 274 different languages and dialects) distributed among 505 indigenous lands in Brazil [9]. The indigenous population can be more vulnerable to the pandemics impact, given their poor social and economic conditions and poor access to health care services, especially populations living in very remote locations. In order to improve the access of indigenous people to health care and adapt the public health care system to their particularities, the Brazilian government created, in 1999, an indigenous health care subsystem, comprised of 34 units named Special Indigenous Health Districts (Supplement 1). Additionally, in 2010, the government created the Special Secretariat for Indigenous Health, which was linked to the Ministry of Health.

Indigenous individuals are under risk of other diseases, such as tuberculosis, which is endemic in Brazils Amazon region as its rates decline worldwide. In the literature, the genetical aspects related with Amerindian genetic ancestry is set as risk factor for tuberculosis [34]. Invasion of indigenous lands is also an old social problem in Brazil due to centuries of predatory agricultural expansion, cattle raising, and even illegal activities, such as wood removal and commercialization, traffic of wild animals, and mining [35]. Such uncontrolled invasion and its consequent harms expose indigenous people to pathogens they are not often in contact with. The indigenous lifestyle can also play a role in the disease spread, as they usually live in collective houses and share utensils. One year after COVID-19 was declared a pandemic, 44,648 indigenous people had been infected, out of whom 605 died and 41,589 recovered (***Table 3***) [9]. Like in the general population, older indigenous adults were the most affected by the COVID-19 pandemic. Culturally, senior individuals are very valued among the indigenous, as they are very representative of the knowledge and culture of each ethnic group, so that their deaths are regarded as great losses. In this context, policies should be urgently adopted by the government, with the support of non-governmental organizations, to reduce the viral spread among the indigenous. Indigenous lands must be kept protected both in order to preserve the indigenous culture and to prevent deforestation of preserved areas [35,36]. Of note, COVID-19 can be devasting for specific indigenous ethnic groups whose populations are smaller. An example is the Juma tribe, which lost its last individual, an 86-year-old man who died after developing severe complications of the disease.

**Table 3:** Distribution of indigenous people affected by COVID-19. Suspected, confirmed, recovered cases, and deaths distributed by the special indigenous health district (dSEI) on March 12, 2021 [9].


dSEI	SUSPECTED CASES	CONFIRMED CASES	ACTIVE CASES	CLINICAL CURE (RECOVERED CASES)	DEATHS

Alagoas and Sergipe	18	331	13	311	5

Altamira	0	1,713	3	1,707	2

Alto Rio Juru	0	863	8	844	10

Alto Rio Negro	34	2,234	144	2,063	25

Alto Rio Purus	0	638	9	621	7

Alto Rio Solimes	0	2154	25	2074	47

Amap and Norte do Par	25	978	37	934	5

Araguaia	0	346	8	331	7

Bahia	18	932	70	852	8

Cear	90	1,092	113	969	8

Cuiab	32	1,301	56	1,221	24

Guam-Tocantins	12	1,509	8	1,481	17

Interior Sul	103	2,647	143	2,456	46

Kaiap do Mato Grosso	9	1,000	0	994	5

Kaiap do Par	29	1,222	0	1,177	9

Leste de Roraima	22	3,855	238	3,553	56

Litoral Sul	7	1,279	3	1,257	17

Manaus	28	1,136	2,252	962	16

Maranho	0	1,687	1,042	1,654	27

Mato Grosso Do Sul	0	4,261	25	4,143	85

Mdio Rio Purus	0	517	0	512	5

Mdio Rio Solimes and Afluentes	6	765	22	730	11

Minas Gerais and Esprito Santo	14	580	39	534	6

Parintins	45	596	11	570	12

Pernambuco	17	624	4	607	10

Porto Velho	23	1,344	31	1,301	11

Potiguara	2	709	1	704	4

Rio Tapajs	0	2,016	47	1,950	16

Tocantins	1	1,176	0	1,162	10

Vale do Javari	0	822	0	818	2

Vilhena	70	899	0	883	15

Xavante	1	908	20	832	50

Xingu	103	1,029	278	718	16

Yanomami	9	1,485	807	664	11

**Total**	**718**	**44,648**	**2,309**	**41,589**	**605**


Other ethnic groups in Brazil should be looked at more carefully, as there is evidence of increased mortality in Pardo and Black individuals, and individuals who live in the North region as a whole [37,38]. These account for the largest fraction of poor individuals in Brazil [39]. Such a socioeconomic disparity contributes to low access to health care, diagnosis, and treatment, which is likely to put them at a higher risk of getting COVID-19 and die of it. In a pandemic scenario, federal and state government measures are urgent and crucial in order to screen these vulnerable groups for COVID-19, curb the viral spread within these populations and facilitate their access to health care services.

### 3.4. Public and private health care systems and the management of the COVID-19 outbreak in Brazil

There are two types of health care services in Brazil, the main of which is the public service, administered by Brazils Unified Health System (SUS), which covers all the Brazilian territory and any and every Brazilian citizen has access to, with no direct costs. The other type is the private health care service, which is individually or collectively paid and acts in a supplementary manner, being regulated by Brazils National Agency of Supplementary Health Care (ANS). It is a legal obligation of private health care providers to refund SUS when any of their users is served by the public health care service for a procedure that is covered by the provider [40].

The private health care service in Brazil is marked by restrictions and disparities, both in its operation and in its access by the population. Currently, 14 companies have 40% of the market and only 2025% of the population have access to private health care assistance. Within this part of the population, 70% reside in the Southeast region. Such disparities reflect in the ICU bed capacity. According to data of the Brazilian Society of Intensive Care Medicine, there are 45,848 ICU beds in the country, out of which 22,844 are available for SUS patients, corresponding to an average of 2.2 total beds per 10,000 inhabitants, and only 1.4 SUS beds per 10,000 inhabitants. However, this number is lower in some regions, especially in the North (Amazonian) and Northeast regions [41]. It is problematic that 21.5% of the ICU beds registered in the SUS are originally private and about 64% are offered by philanthropic entities, meaning that the number of beds offered by the public service itself is even lower. This happens because hospitals in Brazil can simultaneously link up with the public and private health care networks. Also, approximately 31% of the ICU beds in Brazil are destined to private health care. A Fiocruz report shows that, although being accessed by a minor part of the population, the private health care network has a larger amount of ICU beds available per users (62.6 per 100,000 users) than does the SUS (13.6 per 100,000 inhabitants) [12]. This number can be even smaller, as indicated by the Institute of Studies for Health Policies, which reports an average of 7.1 beds per 100,000 inhabitants. Still according to this report, the ICU capacity should at least have been doubled in 53% of the Brazilian territory in order to prevent the public health care system to collapse by the beginning of the COVID-19 outbreak in the country [12].

**Figure 4:**
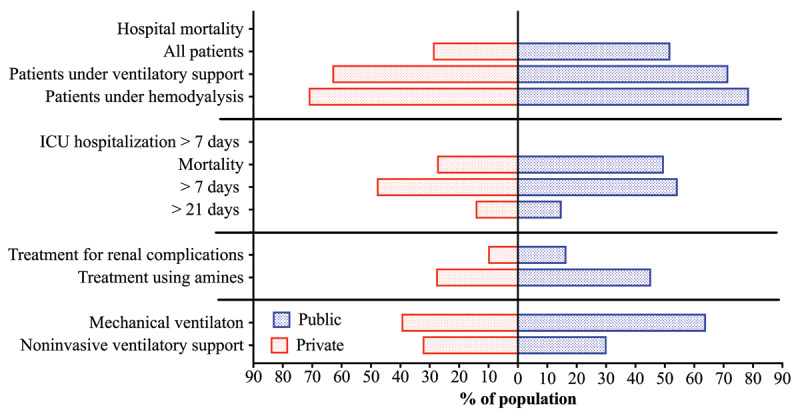
Differences between the Brazilian Private and Public health care systems from 01 March 2020 to 10 March 2021; ICU (Intensive Care Unit). The data were retrieved from Registro Nacional de Terapia Intensiva. [42].

There is also a great disparity between patients with COVID-19 treated in public and private health systems (***Table 6*** and ***Figure 4***). According to a research conducted by the Intensive Medicine Association of Brazil (AMIB) from March 1, 2020, to March 10, 2021, 106,546 patients with COVID-19 were hospitalized, 74,405 (69.83%) of whom in the private health care system and 32,141 (30.16%) in the public health system. Individuals in the private health care system needed more noninvasive ventilatory support than the ones in the public health care system (32.40% vs. 30.20%) [42]. This may be explained by the higher proportion of patients with COVID-19 who needed mechanical ventilation in the public health system (64.00% vs. 39.60%). Individuals in the public health care system also needed mechanical ventilation for less time when compared to individuals from private (11.5 days vs. 14 days), which is possibly due to a higher mortality rate, both in ICU (49.70% vs. 27.50%) and in-hospital (51.90% vs. 28.90%) [42]. It is clear the private health system has a better infrastructure for COVID-19 management than the public health care system; however, the latter is used by most Brazilian citizens, who are not receiving a suitable health care. Increasing the investment in the public health care system is therefore imperative for the Federal Government, in order to decrease COVID-19 mortality.

The main consequence of these disparities in a pandemic setting is that the occupation of ICU beds is lower in private health care units than in the public ones, yielding several idle beds. Solving this issue becomes more difficult in Brazil, as most states in the country do not inform the data about the occupation of ICU beds in the private setting. In some cases, such as the states of So Paulo and Rio de Janeiro, the data concerning public and private networks are merged [43]. There is also no legislation that oblige private health care providers to inform such data. This scenario led the Brazilian National Health Council (CNS) to recommend the Ministry of Health, State and Municipal health authorities to implement a single policy for occupation of ICU beds, which should be made upon demand and regardless of whether these beds are located in public or private units [9], similarly to what has been done in France, Italy, Spain, and Ireland, for instance [44]. A law project was also proposed that sets the mandatory use of beds located in private units by the SUS for the single purpose of admission of patients with ARDS with suspected or diagnosed COVID-19, in addition to the obligation of private health care providers of informing the number of available beds [45]. However, private entities are against this policy and would rather have the government handle the costs of private ICU beds [43]. Public-private partnerships are seen as an interesting alternative in this regard and have been set in the cities of So Paulo and Rio de Janeiro, where private companies, including (but not limited to) health care providers handled the costs of temporary ICU facilities in public spaces of these cities, such as parks and football stadiums [46]. Still, it is worrisome that the current scenario allows private hospitals to deny participating on joint efforts for breaking the advancement of COVID-19, and most private hospitals merely end up making donations of minimal resources.

Within the universe of the private health care network, there are several criticisms of attorneys and consumer protection entities about the lack of austerity of the ANS with services of this nature. About 80% of private health care plans are collective or part of the benefit package offered by companies or institutions to their employees. Also, individual and family plans, which would be susceptible to a more severe control, have become rare. This situation opens gaps for private health care companies to charge a lower price for this kind of plan and to offer services with a lower quality than the standards recommended by the ANS, which is critical in a pandemic setting like COVID-19, where disorganization, insufficient staff board and poor management become more evident [47]. This was the case for a private health care company that offers individual and family plans, in the city of So Paulo. Mainly focused on older adults, this company faced a substantial increase in the demand for ICU admissions shortly after the beginning of the COVID-19 outbreak in the city and is currently under investigation for concealment of COVID-19 cases in its hospitals [48].

### 3.5. The SARS-CoV-2 RT-PCR assay at Brazil: A mistake made in the management of public health

In Brazil, as stated at the literature, there are different moments associated with the diagnosis and data sharing for COVID-19 diagnosis [49]. However, it is evident that Brazil needs to optimize the diagnosis by SARS-CoV-2 RT-PCR, including for health professionals, as many of them are set as suspicious cases due to underdiagnosis [50]. According to the Ministry of Health, Brazil performed a total of 14,725,192 SARS-CoV-2 RT-PCR tests, most of which (about 39%) were performed in the Southeast region (***Table 2***). The Midwest region has the lowest number of tests, most of which are performed in the Federal District, even though the Federal District is the least populated region within the Midwest [9]. The fact that the Federal District is the federal governments seat is likely to be the reason for this. As for the other regions, 2,887,556 tests have been performed in the South region, 3,718,896 in the Northeast Region, and 1,440,140 in the North region (***Table 2***). The lack of tests in the North region is worrisome and can be explained by the difficulty in delivering tests to remote locations in this region, which is the most underdeveloped region in Brazil.

To date, the Brazilian government has spent around R$ 750 million (approximately US$ 134 million) with SARS-CoV-2 RT-PCR tests. Although Brazil has more RT-PCR tests (14,725,192) than quick blood- and antibody-based tests (8,836,305), especially those based on lateral-flow immunoassays (LFIA), performed so far (***Table 2***) [9], only by mid-late 2020 the Federal government started to increase the investment in RT-PCR, meaning that quick tests were most made in the beginning of the pandemic, even though the gold standard for COVID-19 diagnosis is RT-PCR [51]. Quick tests basically rely upon the detection of SARS-CoV-2-specific IgM and IgG, which typically take 1014 days for seroconversion [52,53]. Although antibody-based tests can be appropriate for surveillance studies [54], they do not have the capacity of early diagnosis that SARS-CoV-2 RT-PCR does. Also, a metanalysis found that antibody-based tests were not very well assessed so far and have considerable variation in sensitivity and specificity. LFIA-based tests are of particular concern, as commercial LFIA kits showed a pooled sensitivity of 65% (ranging from 49% to 78.2%), meaning at least 21.8% of false-negative results [55].

Notwithstanding with the insufficient testing, the slow delivery of tests is also worrisome in Brazil. In April 2020, the Ministry of Health announced the purchase of 46.2 million COVID-19 tests, out of which 24.3 million were SARS-CoV-2 RT-PCR tests [9]. Out of 24.3 million, only 8.8 million (36%) were delivered to the Ministry of Health until June, 3.2 million (36%) out of which had been distributed among the Federation states. Other 14.5 million fast antibody-based tests had not been delivered back then and there was no detailed information about these tests in the Ministry of Health information channels [9]. As for SARS-CoV-2 RT-PCR, this scenario can be explained by the difficulty to purchase basic components of the test, due to the high market demand, increase in the price of materials and equipment to perform the RT-PCR, low equipment availability, low number of qualified people available to run the tests, low number of centers and laboratories with infrastructure to run them, and difficulties in the transportation of samples to places where the test can be performed [50,56].

The WHO has emphasized the importance of massive testing, which is the main measure for tracking the viral spread and breaking the SARS-CoV-2 transmission, as about 30% of infected individuals are asymptomatic and can spread the virus unintentionally [57]. The lack of a well-set testing policy, along with the slow distribution of tests across the country makes COVID-19 very difficult to be handled in Brazil. In a country where social distancing is not a possibility in many locations, mass testing is more than essential to prevent a higher number of deaths due to the disease than the one the country already has.

### 3.6. The Brazilian President: A positive case for COVID-19

The Brazilian President, Jair Messias Bolsonaro, has tested positive for COVID-19 on July 14, 2020 [58]. President Jair Messias Bolsonaro first announced his diagnosis on July 7, 2020, after underplaying the severity of the COVID-19 pandemic several times, even after Brazils outbreak became the worst in the world outside the USA. First Lady Michelle Bolsonaro also tested positive for COVID-19, as well as her 80-year-old grandmother, who underwent intubation after developing severe symptoms of the disease and died [59]. Other members of the government who tested positive to COVID-19 include Marcos Pontes (Minister of Science and Technology), Onyx Lorenzoni (Minister of Citizenship), Milton Ribeiro (Minister of Education), Augusto Heleno (Minister of the Institutional Security Office), Bento Albuquerque (Minister of Mines and Energy), Otvio Rego (President Jair Messias Bolsonaros spokesman), and Fabio Wajngarten (Ministry of Communications Executive Secretary) [60].

Several sayings and attitudes by President Jair Messias Bolsonaro had a strong backlash over the globe and were very criticized by many specialists in the field and by the national and international media. Those include President Jair Messias Bolsonaro claiming that his good physical condition and athlete history would prevent him from developing severe symptoms in case he would be infected with SARS-CoV-2 [61]. The President also pushed back against lockdown measures in the Federation states and in the cities, incentivizing most of the population to keep their normal lives. Scenes of President Jair Messias Bolsonaro circulating without a facial mask in public spaces were very frequent [62], including when he was knowingly infected, when the President was seen driving a motorcycle without either facial mask or individual protection equipment and talking to street sweepers who were working in the Federal District capital, Braslia [63]. Before his COVID-19 diagnosis was confirmed, President Jair Messias Bolsonaro frequently attended events in which his group of supporters agglomerated in public places demanding the end of the social isolation measures. Politically, President Jair Messias Bolsonaro has shown a faithful submission to former USA President Donald Trump, who also insisted on underplaying the pandemic when it was declared and continued to do so regularly. Some terms were used by each or both to refer to COVID-19, such as Chinese virus, hoax, little flu, and mediatic exaggeration [64,65]. Both executive leaders also took COVID-19 to the political field, with statements that flirt with conspiracy theories, arguing that the diseases impact is overestimated, both by the media and opposition politicians and parties, to degrade the governments image [66,67].

Moreover, the Brazilian population is influenced by massive fake news involving COVID-19, many of which are powered by President Jair Messias Bolsonaro. Common stories in fake news are that the number of COVID-19 cases and deaths are inflated; drugs that were ruled out as possible treatments for COVID-19 are efficient, such as hydroxychloroquine and ivermectin; images of empty coffins being buried have been used to scare the population about the COVID-19 hoax; and social isolation and use of facial masks are not efficient against the viral spread [68]. Interestingly, a study comprising 104 cities with 70,000 or more inhabitants in the So Paulo State found an inverse correlation between the rate of adherence to social isolation and the number of votes to President Jair Messias Bolsonaro (% of population in the first and second round of election; ***Table 4***). The social isolation index was computed by So Paulo government [69].

**Table 4:** Spearman correlation between adhesion to social isolation measures (% of population) during the COVID-19 pandemic and number of votes to President Jair Messias Bolsonaro (% of population in the first and second round of election) [69].


PHYSICAL ISOLATION	CORRELATION	FIRST ROUND OF ELECTIONS	SECOND ROUND OF ELECTIONS

First month (% after 30 days of first confirmed case)	Correlation coefficient	0.299**	0.197*

	p-value	0.002	0.046

Minor adhesion (%)	Correlation coefficient	0.280**	0.175

	p-value	0.004	0.076

Major adhesion (%)	Correlation coefficient	0.293**	0.218*

	p-value	0.003	0.027


### 3.7. From phosphoethanolamine to chloroquine and ivermectin: President Jair Messias Bolsonaros miraculous pills

The belief of President Jair Messias Bolsonaro in miraculous drugs is not new and dates to 2016. Before that year, Carlos Witthoeft, a citizen of the town of Pomerode, in the state of Santa Catarina, was arrested in June 2015 by the state police, after illegal production of a pharmacological component named phosphoethanolamine, which allegedly had the potential of curing cancer. Witthoeft claimed that the drug had a pivotal role in curing his mothers cancer in only 18 days back in 2007. He was instructed by Gilberto Chierice, a professor at the University of So Paulo (USP) So Carlos. Chierice was forbidden both by USP So Carlos and by the So Paulo state court to produce and distribute phosphoethanolamine, which caused a race of its users to the states judiciary system to claim access to it. The phosphoethanolamine ban was then revoked by the National Supreme Court, which authorized its use as a palliative treatment option for cancer, but not really as a medication [70]. Phosphoethanolamine was extremely endorsed by, at the time deputy, President Jair Messias Bolsonaro in his social medias, and the President co-authored a law project (n. 13.269, 2016) that predicted the regulation and authorization of the use and distribution of phosphoethanolamine [71]. The law was sanctioned by, at the time, President Dilma Rousseff. In 2017, one year after the laws approval, phosphoethanolamine still would not show antitumoral efficacy [72]. The phosphoethanolamine hype started to fade out and, nowadays, the drug is barely remembered as an alternative to cancer treatment by the medical and scientific communities, government, and society.

When the number of COVID-19 cases started to show a sharp increase in Brazil, President Jair Messias Bolsonaro stated A miracle drug was on hand. The drug was hydroxychloroquine, used in the treatment of malaria and chronic inflammatory diseases, especially systemic lupus erythematosus (SLE) and rheumatoid arthritis. The President Jair Messias Bolsonaros excitement about hydroxychloroquine had its origin in the drugs promotion by former USA President Donald Trump. After learning about the alleged benefits of the drug in patients with COVID-19 reported by a French study [73], which was criticized in the very same journal that published it and happened to have severe methodological gaps that were admitted by the corresponding author [74,75]. President Donald Trump claimed that hydroxychloroquine could be a game-changer in the fight against SARS-CoV-2. Reinforcing Trumps statements, President Jair Messias Bolsonaro also recommended hydroxychloroquine as a treatment for COVID-19 in a live address [77]. The Brazilian president excitement about hydroxychloroquine was boosted by a study performed in Brazil and sponsored by a private health care provider, which suggested the use of the drug for COVID-19 treatment. However, like the French report Trump based upon, this study was strongly criticized by the scientific community due to several methodological issues, which included strong risks of bias. As a matter of fact, the study made in Brazil was suspended by the Brazilian National Council on Research Ethics due to fraud suspicions [78].

The criticisms of the scientific community did not stop President Jair Messias Bolsonaro from promoting the use of hydroxychloroquine for COVID-19 treatment. In the State of Rio de Janeiro, the drug started to lack in the stocks of pharmacies, after an uncontrolled search by people. President Jair Messias Bolsonaros insistence on the this costed the loss of two Ministers of Health in less than one month, as we will address in the next section (3.8). To date, the hydroxychloroquine use for COVID-19 treatment is authorized by the Ministry of Health in specific cases and through informed consent. President Jair Messias Bolsonaro also promoted Ivermectin as a treatment for COVID-19 [79]. This vermifuge is used to treat several diseases caused by ectoparasites, such as *Strongyloides stercoralis* [80], and is not effective against COVID-19 [81], as stated by its own manufacturer [82].

President Jair Messias Bolsonaro is a fierce defender of the early COVID-19 treatment, even though there are no studies showing any evidence that such an approach exists. Immunosuppressant drugs, such as dexamethasone, which have been proven useful to treat severe COVID-19 and help to decrease mortality [6], have not been promoted by President Jair Messias Bolsonaro at all. Notably, under President Jair Messias Bolsonaros command, the availability of chloroquine and hydroxychloroquine has strikingly increased in Brazil, both by massive production in the laboratories of the Brazilian armed forces and by importing these drugs from abroad with no binding [9]. A total of 5,416,510 tablets of chloroquine and 481,500 tablets of hydroxychloroquine have been purchased by the federal government in the last year, corresponding to R$ 200,000 spent with chloroquine, while the total expenses with hydroxychloroquine are not informed by the Ministry of Health official platforms [9]. Also, the federal government expend a huge value for advertisements highlighting the important of early treatment and to distribute chloroquine and hydroxychloroquine during the year 2020. Concomitantly, essential drugs for treatment of severe and critical COVID-19 cases, such as sedatives used in orotracheal intubation, are missing in hospitals [9].

SARS-CoV-2 RT-PCR tests are also important to determine the positive cases for infection and to give the opportunity for a prompt response to the viral spread. Also, these markers demonstrated a positive Spearman correlation with (i) confirmed COVID-19 cases (CC: 0.849, *p* < 0.001); (ii) death due to COVID-19 (CC: 0.802, *p* < 0.001); (iii) lethality (CC: 0.335, *p* < 0.001); and (iv) deaths due COVID-19 per one million of inhabitants (CC: 0.260, *p* = 0.006). Finally, the number of SARS-CoV-2 RT-PCR tests per one million of inhabitants was positively correlated with and confirmed COVID-19 cases per one million of inhabitants (CC: 0.533, *p* < 0.001) and deaths due to COVID-19 per one million of inhabitants (CC: 0.274, *p* = 0.004) (***Table 5***). Although medical and pharmaceutical sciences are not President Jair Messias Bolsonaros areas of expertise, the President insists on spreading fake news about the efficacy of knowingly inefficient drugs in one of the most delicate moments in Brazils history, raising concern about President Jair Messias Bolsonaros real interests in their promotion and use by people. The situation is aggravated by President Jair Messias Bolsonaros solid support base, which endorses the use of these drugs. After all, the phosphoethanolamine episode in 2016 was a preview of the chloroquine-hydroxychloroquine-ivermectin hype of 2020, which have no proven efficacy in the treatment of COVID-19 and have several adverse effects [83,84,85,86,87,88,89]. However, the backlash was not so high back then as it was in 2020, and the phosphoethanolamine case did not draw the attention of the Brazilian population, in general. One might remind the quote by Edmund Burke: Those who dont know history are doomed to repeat it.

**Table 5:** Correlation between number of vaccine shots, SARS-CoV-2 RT-PCR tests and confirmed COVID-19 cases, deaths due COVID-19, and lethality rate. Data retrieved from 112 countries and territories.


MARKERS	DATA	VACCINATIONS DOSAGES	VACCINATION PER 100 PEOPLE	NUMBER OF RT-PCR TESTS	NUMBER OF RT-PCR TESTS/1M INHABITANTS

Confirmed COVID-19 Cases	CC	**0.832**	0.051	**0.849**	0.075

	P-value	**<0.001**	0.593	**<0.001**	0.438

Death due COVID-19	CC	**0.786**	0.104	**0.802**	0.138

	P-value	**<0.001**	0.274	**<0.001**	0.152

Lethality	CC	**0.284**	**0.210**	**0.335**	**0.251**

	P-value	**0.002**	**0.026**	**<0.001**	**0.008**

Confirmed cases/1M	CC	**0.235**	**0.418**	0.164	**0.533**

	P-value	**0.013**	**<0.001**	0.087	**<0.001**

Death/1M	CC	**0.303**	**0.209**	**0.260**	**0.274**

	P-value	**0.001**	**0.027**	**0.006**	**0.004**

Number of RT-PCR tests	CC	**0.849**	0.033		0.095

	P-value	**<0.001**	0.731		0.327

Number of RT-PCR tests/1M Inhabitants	CC	0.023	**0.645**	0.095	

	P-value	0.812	**<0.001**	0.327	


CC, coefficient correlation; 1M, one million; RT-PCR, real time polymerase chain reaction. The Supplement 2 demonstrated the data used to perform the correlation. The information for COVID-19 Cases, Death due COVID-19, Lethality and SARS-CoV-2 RT-PCR was obtained using WorldOMeter. Cases of Coronavirus in Brazil. 2021. Accessed on March 11, 2021. Available at *https://www.worldometers.info/coronavirus/country/* [10]. The reference for the number of vaccinations dosages was retrieved from Coronavirus (COVID-19) Vaccinations on March 11, 2021 at *https://ourworldindata.org/covid-vaccinations*.

**Table 6:** Major differences between in Brazilian private and public health care (March 01 2020 to March 10 2021) [42].


MARKERS	ALL	PRIVATE	PUBLIC

COVID-19 new hospitalizations	106,546	74,405	32,141

Ventilatory support			

Noninvasive ventilatory support	31.8%	32.4%	30.2%

Mechanical ventilation	46.9%	39.6%	64.0%

Mechanical ventilation (days)	13	14	11.5

Amines	33.0%	27.8%	45.3%

Kidney Support	12.0%	10.1%	16.5%

ICU hospitalizations (days)	12.2	11.9	12.7

> 7 days	49.9%	48.0%	54.4%

> 21 days	14.5%	14.4%	14.9%

ICU mortality	34.1%	27.5%	49.7%

Hospital mortality			

All patients	35.6%	28.9%	51.9%

All patients with no ventilatory support	9.2%	7.4%	16.6%

All patients with ventilatory support	66.6%	63.1%	71.6%

Dialysate patients	74.2%	71.2%	78.6%

ICU, intensive care unit.

### 3.8. An unhealthy Health Ministry

During the onset of the COVID-19 pandemic, Brazil had three ministers of health. Luiz Henrique Mandetta, the first one, took office on January 01, 2019 staying until April 04, 2020. Mandetta is a medical doctor who saw his popularity increase when the outbreak reached Brazil, after being active in several media vehicles, both talking about the severity of the COVID-19 situation and announcing the measures taken by the Ministry of Health aimed at controlling the SARS-CoV-2 spread, which included the recommendation of social isolation, quarantine, personal hygiene, and use of personal protective equipment, lockdowns, expansion of the RT-PCR testing capacity, and creation of temporary ICU facilities. However, Mandetta resigned after several conflicts with the government. His successor, Nelson Teich, also a medical doctor, took office on April 17, 2020 and resigned on May 15, 2020. During his short period as a Minister of Health, Teich had the burden of reconciling the guidelines recommended by the Brazilian government with those recommended by the WHO. However, Teich had several divergences with President Jair Messias Bolsonaro, and saw his authority as a minister be disrespected, including in an emblematic episode, when, during a live interview, he learned that President Jair Messias Bolsonaro, gave his own recommendations concerning the COVID-19 situation to the Brazilian population without prior consultation with the Ministry of Health. Teich also saw himself pressured to approve the use of chloroquine and hydroxychloroquine as treatments for COVID-19, which he did not agree with. Teich then resigned after less than one month in charge.

On May 15, 2020, Eduardo Pazuello, a general of the Brazilian Army, was nominated Brazils interim Health Minister, and was made the official health minister in September. General Pazuellos background includes a parachutist course but no activities related to health care. Pazuello had previously acted as the Ministry of Health executive secretary and, upon President Jair Messias Bolsonaros request, coordinated the transition period between Mandettas and Teichs administrations. During Pazuellos mandate, little has been done in order to face the pandemic. When health specialists and authorities stressed the importance of mass testing, Pazuello said that the testing capacity in Brazil would be increased, but no deadlines for this were set, and the access of the population to COVID-19 diagnosis is still difficult nowadays. Although Pazuello was introduced as a specialist in logistics, during his term, about seven million SARS-CoV-2 RT-PCR tests were found to be retained in So Paulo by the Ministry of Health that were about to expire and had a cost nearly to R$ 290 million. During his inauguration ceremony, Pazuello gave a speech that proved to be in line with what had been defended by President Jair Messias Bolsonaro, including the so-called early COVID-19 treatment, which was put into practice afterwards, when Pazuello did what his predecessors refused to do and signed a technical opinion that set the guidelines for the use of hydroxychloroquine for COVID-19 treatment. Pazuello also appeared, on several occasions, without wearing a mask alongside authorities [90]. However, nothing was more remarkable during Pazuellos management than the lack of a vaccine policy, as better detailed in section 3.13, later in the present article.

In January 2021, the city of Manaus (Amazonas state), thought to have achieved herd immunity after the first COVID-19 wave [92], faced a devastating second wave of the disease, with a dramatic increase in the number of cases, hospitalizations, and deaths. Pazuello was notified about the lack of oxygen cylinders in hospitals of Manaus and about the risk of collapse. Little was done by the Health Ministry in this regard and Pazuellos conduct in the Manaus crisis became an object of enquiry in the Brazilian supreme court, and Pazuello is currently being investigated for omission. The purchase of drugs without proven efficacy against COVID-19 as an alternative to face the health crisis in Manaus is also under investigation. As a new collapse of health care services became a reality nationwide again, and the country achieved more than 2,000 daily deaths due to COVID-19, Pazuello alleged that Brazils health system is surely impacted, but it has not collapsed and never will. After 10 months as a Health Minister, Pazuello resigned on March 14 2021 [90].

After Pazuellos resignation was made official, President Jair Messias Bolsonaro interviewed medical doctor Ludhmila Hajjar, candidate to the Health Ministry as per indication by the high command of the Brazilian congress. Hajjar is known to be pro-science and against the prescription of treatments with no proven efficacy against COVID-19. However, due to technical differences, Hajjar did not accept the post. President Jair Messias Bolsonaro then invited medical doctor Marcelo Queiroga, president of the Brazilian Society of Cardiology, to occupy the post, which he accepted. In 2020, the Brazilian Society of Cardiology, presided by Queiroga, recommended that chloroquine, hydroxychloroquine, and azithromycin were not used to treat COVID-19. However, after the backlash, the Society and the Ministry of Health released a joint note, opening the possibility for COVID-19 patients to use these drugs upon written informed consent and offering to monitor any resulting side effects [93]. Also, Queiroga is a defender of social isolation measures and use of facial mask [94].

Decision making to implement public health policies must be effective and guided by the governments health representative, especially during events like the COVID-19 pandemic. International recommendations be followed with minor adaptations according to local characteristics. However, in Brazil, one minister of health resigned after a disastrous management, and two others resigned after being pressured by the countrys president to implement measures that do not follow the international and scientific-based recommendations to control the increase in the number of COVID-19 cases and to treat infected patients, including the use of chloroquine and hydroxychloroquine. President Jair Messias Bolsonaros attitude and the lack of a fixed minister of health not only impair the implementation of appropriate measures to handle the outbreak, but also lead to a decreased adherence of the population to the safety measures.

### 3.9. How much can we pay to breathe? The cost of mechanical ventilators in Brazil

Respiratory failure contributes to worse prognosis in COVID-19, so that oxygen therapy and ventilatory support, including mechanic ventilation with positive pressure, are crucial to treat severe cases of the disease in order to avoid airway collapse and to optimize hemostasis [95]. Due to a high demand for mechanical respirators caused by the COVID-19 outbreak, the government had to purchase a great number of new equipment. Until the first fifteen days of July 2020, 7,994 pulmonary ventilators were delivered across the country via Ministry of Health. The criteria for distribution of these respirators considered the structure of a given service and the presence of specialized teams to operate them, which, in turn, had the participation of the National Council of Health Secretaries (CONASS) and the National Council of Municipal Heath Secretariats (CONASEMS).

Until July 2020, the Ministry of Health had signed five contracts with Brazilian companies so they could start producing 16,252 respiratory ventilators, out of which 6,500 were produced by Magnamed, with a cost of R$ 332.5 million (US$ 60.219 million); 4,300 by Intermed, with a cost of R$ 258 million (US$ 48.175 million); 3,300 by KTK, with a cost of R$ 78 million (US$ 14.564 million); 1,202 by Leistung, with a cost of R$ 72 million (US$ 13.444 million); and 950 by WEG, with a cost of R$ 57 million (US$ 10.643 million) [9]. The mechanical ventilators were distributed to the states and the Federal District as shown in ***Table 2***. At the same time, some Brazilian states also purchased mechanical ventilators separately, which a large difference seen among the paid values [96]. The state of Mato Grosso paid R$ 44,000 per unit for ventilators made in Brazil and R$ 61,700 per unit for imported equipment. In contrast, the State of Roraima paid R$ 215,4 per unit. Some of these cases under investigation for over-purchase and diversion of public money. In the State of Santa Catarina, a scandal came to light revealing that the states government ordered the purchase of 200 respirators, with a final cost of R$ 33 million by using fake data and quotes. Five people were arrested so far, under the accusations of embezzlement, active and passive corruption, and money laundering. The case was assigned to the Brazilian Supreme Court after Santa Catarina governor Carlos Moiss became a suspect [97].

### 3.10. The availability of drugs to perform intubation in patients with COVID-19

On July 3, 2020, a public hearing was held in the Brazilian Congress. At the hearing, the CONASS adviser, Heber Dobis, participated in a debate on pricing and order of sedatives and other drugs to perform intubation of patients with severe or critical COVID-19, promoted by the External Commission for Actions against coronavirus. At least three weeks ago, the health departments of several Brazilian states were facing difficulties in supplying drugs used in the process of orotracheal intubation for mechanical ventilation. A survey performed by the CONASS and answered by the health secretariats of 25 states found that muscle blockers were lacking in 24 states, and 11 other essential drugs were lacking in more than half of the states. The lack of medication for inducing sedation, anesthesia, and muscle relaxation was described as extremely serious, which could make mechanical ventilation unfeasible [98].

Subsequently, the CONASS mapped the stocks of medicines in health units in the states and found that several states were facing shortages of essential medications or risk of shortages in the coming days from the end of June. The State of Mato Grosso had the highest number of missing items (13), followed by the states of Cear and Maranho (12), Amap and Tocantins (11), Rio Grande do Norte (10), Roraima, Amazonas, and Bahia (9), and Pernambuco (8). The States of Alagoas, Minas Gerais, Paran, Piau, Santa Catarina, and Sergipe are fully supplied. Additionally, nine drugs were only sufficient to meet the demand expected for the next five days in Mato Grosso. In Pernambuco, eight drugs were missing and nine were due to expire in a week. So Paulo, the COVID-19 epicenter in Brazil, had only one medication unavailable, but 14 other drugs were available, enough for only five days [99].

### 3.11. Health care professionals affected by COVID-19 in Brazil

**Figure 5:**
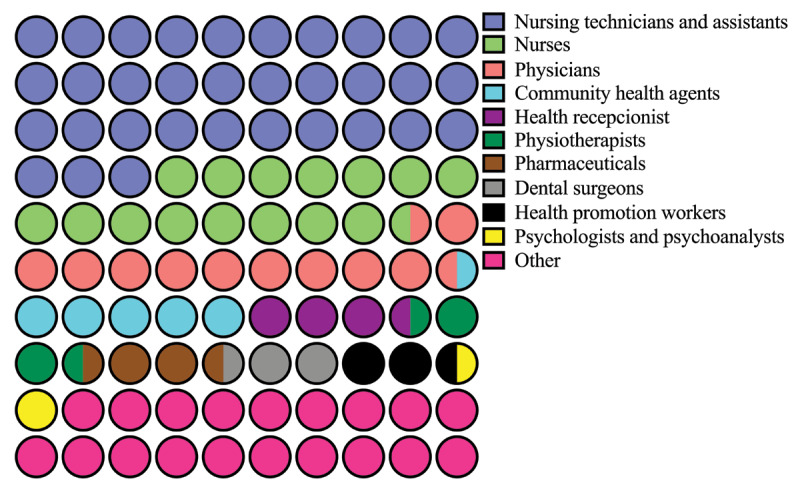
Health care workers affected by COVID-19 in Brazil after one year of pandemic. The data are given as percentages. Source: Brazil, Ministry of Health. 44^th^ and 52^nd^ Special Epidemiological Reports, 2020/2021. Accessed on 17 March 2021. Available at *https://coronavirus.saude.gov.br/boletins-epidemiologicos* [9].

Health care workers have a higher risk of being infected with SARS-CoV-2 [100]. A study with 32,583 Chinese patients with COVID-19 [101] found that health care workers represented 4.6% of confirmed cases. Overall, the COVID-19 incidence seems to be three times higher in health care workers, if compared to the general population [102]. In Brazil, one year after pandemic was declared, 481,285 COVID-19 cases had been confirmed among health care workers, with nursing assistants being the most affected group (33.2%), followed by nurses (15.3%), physicians (11.1%), health care community agents (5.1%), and receptionists of health units (3.9%) (***Table 7*** and ***Figure 5***) [9]. In addition, 470 health care workers died of COVID-19 in Brazil, 153 of whom were nursing assistants, 95 were physicians, and 59 were nurses. In January 2021, the International Council of Nurses reported 2,262 deaths of nurses due to COVID-19 [103]. By the same period, Brazil accounted for at least 30% of the deaths of health care workers due to COVID-19, according to Brazils Federal Nursing Council [104]. These figures may be explained by the lack of personal protective equipment, such as N95 facial masks, and sanitizers in many health care units across the country. Health care workers should be carefully monitored, as they are the key workers in the fight against the outbreak. Also, health care workers with asymptomatic COVID-19 who are not tested are more likely to infect people in health care settings. In fact, massive and continuous testing is crucial to prevent contagion and to reduce the in-hospital SARS-CoV-2 transmission [105,106,107]. Not by chance, health care workers were the first group to be vaccinated against COVID-19 in Brazil and in most countries of the world [24].

**Table 7:** Number of health care worker accompted by SARS-CoV-2 infection during the first year of COVID-19 pandemic in Brazil.


Health care worker	Number of COVID-19 cases until 27 February 2021 (%)

Nursing technicians and assistants	159,786 (33.2%)

Nurses	73,819 (15.3%)

Physicians	53,549 (11.1%)

Community health agents	24,540 (5.1%)

Health receptionist	18,672 (3.9%)

Physiotherapists	14,439 (3.0%)

Pharmaceuticals	13,031 (2,7%)

Dental surgeons	12,958 (2.7%)

Health promotion workers	11,641 (2.4%)

Psychologists and psychoanalysts	7,421 (1.5%)

Other HCW*	91,429 (19.0%)

Total	481,285


* Other HCW accounts for: managers and operations specialists in companies, departments, and health service units, endemic health agents, ambulance drivers, caregivers, health managers, dentistry technician, nutritionists, pharmacy and pharmaceutical manipulation technicians, social workers and home economists, technicians from health laboratories and blood banks, public health agents, biomedical, radiology assistants, attention, defense, and protection workers for people at risk and adolescents in conflict with the law, technologists and technicians in diagnostic and therapeutic methods, work safety technicians, other teaching professionals, health laboratory assistants, veterinarians and zootechnicians, telephone operators, speech therapists, rescuers (except doctors and nurses), physicists, technicians in food production, preservation and quality, physical education professionals, occupational therapists, orthopedists and psychomotricists, piotechnology professionals, teachers, biologists, production, quality, safety and related engineers, biological sciences researchers, electro-electronics and photonics technician working in healthcare, orthopedic immobilization technicians, health and environmental agents, technologists and technicians in complementary and aesthetic therapies, chemistry teachers, photographic and radiological laboratory workers, technicians in orthopedic prostheses, health records and information workers, optics and optometry technicians, food and related engineers, music therapists, art therapists, equotherapists or naturologists, doulas, lay midwives, electricity and electrotechnical technicians, professionals of creative, equotherapic, and naturological therapies, biotechnology support technicians, funeral service workers, osteopaths and chiropractors, bioengineering support technicians, necropsy technicians, and taxidermists. The information was acquired from Ministrio da Sade. 44 e 52 Boletim epidemiolgico especial. 2020/2021. Accessed on 17 March 2021. Available at *https://coronavirus.saude.gov.br/boletins-epidemiologicos* [9].

Keeping health professionals protected against SARS-CoV-2 infection is not only a manner of avoiding in-hospital infection, but also to improve the management of infected individuals, especially those with severe and critical disease, in need of ICU referral and intubation.

### 3.12. Health care investment is crucial to attenuate the COVID-19 impact

Back in 2017, Brazil was among the 10 wealthiest countries in the world, with a gross domestic product (GDP) of US$ 2.063 trillion, still far from the USA (USD 19.485 trillion) and China (USD 12.310 trillion). On the other hand, in terms of health investment, Brazil (9.47% of the GDP) was ahead of China (5.15% of the GDP), but not of the USA (17.06% of the GDP), which had the second highest percentage of the GDP invested in health [8]. Despite having a higher GDP percentage invested in health, Brazil and USA were not as successful as China in the management of the coronavirus crisis. Soon after its local outbreak, the Chinese government implemented stringent measures to curb the viral spread, managed to plateau the number of daily deaths by mid-April 2020 and faced a weak second wave, whereas Brazil and the USA kept seeing their numbers increasing every day [8], had a much worse second wave, and, together, account for nearly 30% of all deaths in the world. Chinas successful policies contrast with how the pandemic was handled in Brazil and in the USA in its beginning, when the main authorities in both countries underplayed its potential, as well as the recommendations given by both local specialists and WHO. In fact, the USA, upon Donald Trumps command, stopped directing funds to the WHO, with the former USA president also accusing the WHO of being under Chinas control [108]. After starting a massive vaccination campaign by late December 2020, the USA did not take long until it saw a noticeable decrease in the number of daily new cases and daily new deaths [10,24].

It is well known that health investment is essential to improve the quality of health services and boost economy [109]. A solid public health system can also facilitate human development [8]. Still, this is not necessarily enough to succeed in a pandemic setting. Reckless actions, such as those taken by Brazil and USA, for instance, can be more harmful to the population during a pandemic than low health investment itself. After the world has seen the success of countries such as China, South Korea, Germany, Denmark, and New Zealand in breaking COVID-19 advancement, it became crystal clear that a high health investment, when associated with a proper conduct of a countrys authorities, is crucial for the fate of any country in such a moment.

Last but not least, Brazils SUS should be valued and strengthened, because it is used by most of the Brazilian population. The SUS still faces numerous issues nowadays, when it comes to its efficiency, most of which can be associated with failures in its decentralization process, poor administration not only by the Federal Government, but also by states and municipalities diversion of funds, corruption, poor transfer of funds coming from taxes, lack of new contracts in view of the current demand for the maintenance of several health services, and scrapping of facilities and equipment, leading to a poor investment in health, which is likely to have contributed to the health care collapse. Considering indirect and direct taxations, Brazil has one of the highest tax rates in the world. However, the way taxes are managed in the country is one of the major complaints of the Brazilian population, which has the perception that benefits coming from taxation are not satisfactory.

### 3.13. The vaccination status in Brazil

Manaus, in the Amazonas State, was the first Brazilian capital city to face a collapse in its health care system during the first wave of COVID-19, reaching an epidemic peak between March and April 2020. In June, the city had a cumulative rate of 52% of SARS-CoV-2 seropositivity, which started to decline in July and August of the same year, suggesting that herd immunity had been achieved [110]. However, Manaus not only faced a devastating second wave of COVID-19 but was also the source of emergence of the P1 lineage of SARS-CoV-2, which has been shown to be more transmissible and able to cause reinfection [111] and became a matter of concern worldwide. Manaus case may have been the last hope of achieving natural herd immunity to infection, an outcome that was implicitly expected by the Swedish health authorities in their anti-COVID-19 strategies in the early days of the pandemic and did not succeed as well [112]. Manaus and Swedens cases make it clear that herd immunity is a distant reality and stress the importance of vaccination to reduce the viral spread. In this scenario, COVID-19 drove the development of next-generation vaccines that entered human clinical testing with unprecedent rapidity [113], some of which are rolling out in many countries, namely mRNA-1273 (developed by USAs Moderna), BNT162b2 (developed by Pfizer and BioNTech), ChAdOx-1 (developed by the Oxford University and Astra-Zeneca), AD26.COV2.S (developed by Janssen Johnson & Johnson) Sputnik-V (developed by Russias Gamaleya Institut), CoronaVac (developed by the Chinese company Sinovac Biotech) and BBIBP-CorV (developed by the Chinese company Sinopharm).

As previously stated, the lack of a vaccination plan was the main mistake by Brazils Health Ministry. After being stocked with active-duty and retired military officials with little or no public health experience, the Health Ministry, under Eduardo Pazuellos command back then, fell asleep at the wheel and failed when efforts were needed to ensure the acquisition of basic supplies, to build up a structure for a massive vaccination, and to set deals with vaccine makers. After being pressured by state governors, Pazuello announced the purchase of 46 million doses of the Chinese CoronaVac, whose production in Brazil was started after setting up a partnership with the Butantan Institute, which is located in the state of So Paulo, governed by Joo Dria. However, on the next day, Pazuello was unauthorized by President Jair Messias Bolsonaro, who alleged that nobody was interested in this Chinese vaccine but Brazil, as Pazuello quarantined after having COVID-19. The government went back on its decision, and Pazuello presented a plan for acquisition of 46 million doses of the Coronavac vaccine until the end of March, which was changed five times until the predicted estimate was reduced to 2225 million doses. In the meantime, it came to light that the pharmaceutical company Pfizer had offered a deal for 70 million doses of its developing vaccine by the Brazilian government to be delivered on December 2020. The negotiation got stuck as the government did not agree with the terms. However, Brazil ended up missing the deadline to give Pfizer an answer and the vaccine doses were never delivered. In February 2021, Butantan Institute president Dimas Covas affirmed that the Ministry of Health declined an offer of 160 million doses of the CoronaVac by Sinovac. Altogether, the doses of the Pfizer/BioNTech and Sinovac vaccines would be enough to vaccinate 50% of the Brazilian population with two shots [90,91].

So far, in addition to the Sinovac-Butantan agreement, two other agreements have been signed in Brazil that predict transfer of technology for vaccine production, one of which between AstraZeneca and Fiocruz, and the other one between the Gamalyea Institute and the State of Parans Institute of Technology [114]. Only the AstraZeneca-Fiocruz agreement was funded by the Ministry of Health. The initial plan predicted 15 million doses to be produced in December 2020 and other 15 million in January 2021. However, by that time, to produce the vaccine, Fiocruz lacked the active ingredient, which needed to be imported. Due to the international competition, the shipment of this ingredient to Brazil was slowed, directly impacting the vaccination plan. In mid-March 2021, the Brazilian government finally reached an agreement for acquisition of 138 million doses of vaccines, 100 million of which will be provided by Pfizer and 38 million by Janssen. The total cost of the operation will be around US$ 1.38 billion; however, the deadline for delivery has not been revealed. Other agreements were announced with AstraZeneca, Sinovac Biotech, and the Covax Facility Consortium (coordinated by WHO), and another agreement is expected with the Gamaleya Institute. The Ministry of Health has affirmed that Brazil will have more than 400 million doses of the vaccine until December 2020 [115,116,117].

As vaccines were under development and most countries in the world were rushing to reach deals with vaccine makers, a skeptical President Jair Messias Bolsonaro stated, in October 2020, in one of his traditional live broadcasts on his Facebook page, that we are not buying vaccines and no Brazilian will be a guinea pig. Back then, Brazils best hope for a short-term vaccination strategy was CoronaVac, which President Jair Messias Bolsonaro continuously attacked, suggesting that it could kill or disable people who take it, with no evidence at all [118]. In an outrageous episode, the CoronaVac Phase III clinical trial was stopped after the death of a volunteer, which President Jair Messias Bolsonaro celebrated as Another victory [119]. The death turned out to be a suicide and the volunteer was actually in the placebo group. Other statements include I am not taking the vaccine. I got infected already and got antibodies. So, what is the point?, Only my dog can be obliged to be vaccinated, Half of the population will not take the vaccine. These kinds of statements are dangerous in a pandemic setting, especially when anti-vaccine movements are rising around the world, including in Brazil, where vaccine hesitancy has been growing in the past five years [120].

Brazil started its vaccination plan on January 18, 2021, and individuals vaccinated in the first phase of the vaccination program included health care workers, older adults aged more than 60 years old or who lives in institutions, and indigenous people [9]. Given the history of successful vaccination strategies and the availability of state-funded facilities for large-scale vaccine production in Brazil, the country should be much better positioned in this regard. However, due to governmental negligence earlier in the pandemic, Brazil has around 12 million doses administered, only 5% of its population vaccinated with at least one shot (***Figure 1***), and only 1.5% vaccinated with two shots so far [24], which places the country behind other emergent nations like Chile and Bahrein in the vaccination rank (Supplement 2); at the same time the country is the epicenter of the pandemic. On the other hand, scientific efforts are seen, such as a Butantan Institutes study, in which 30,000 people in Serrana, a town with nearly 45,000 inhabitants in the countryside of So Paulo, will be vaccinated with the CoronaVac, aiming at a deeper evaluation of the vaccines efficacy and the possible achievement of herd immunity in the town [121].

At present, potentially more lethal lineages of SARS-CoV-2 are circulating around the world and other lineages can appear as the virus undergoes continuous mutations. Massive vaccination is therefore important, not only for individual protection, but also for collective protection, in order to halt the circulation of the virus. Still, vaccines are not supposed to completely sweep an infection out. They rather reduce the odds of getting severely ill due to the infection. This can be shown by Israels example, where nearly 60% of its population and observing was vaccinated and a sharp decrease in the death and hospitalization rates followed [24]. This is the path Brazil, and all other countries, must follow in order to prevent further health care collapse and to turn COVID-19 into one more endemic disease. In the meantime, stringent social isolation measures are needed, even as vaccination progresses worldwide.

Nevertheless, a positive Spearman correlation was found between vaccination dosages and COVID-19 markers such as COVID-19 cases, number of deaths due COVID-19, number of SARS-CoV-2 RT-PCR tests, and lethality in a statistical analysis from 112 countries or territories. The same occurred between vaccination per 100 people and COVID-19 cases or deaths due COVID-19 per one million inhabitants. Unfortunately, a negative correlation was found between vaccination per 100 people and SAR-CoV-2 RT-PCR tests per one million of inhabitants with lethality, which leads to the belief that vaccination per 100 people and number of SARS-CoV-2 RT-PCR tests per one million inhabitants is lower in regions with higher lethality rate (***Table 5***).

## 4. Highlights

One year after COVID-19 was declared a pandemic, Brazil had the second higher number of cases and deaths, and the highest number of daily deaths due to the disease.
Lack of massive testing, non-stringent and ineffective collective health policies, poor management of the public health care system, and political instability were the main drivers of the countrys flawed management of the COVID-19 advancement.
Due to previous governmental negligence, massive vaccination in Brazil will be a challenge.
Anti-science and sabotaging actions by government had a pivotal role in the countrys current situation.
Brazil has a large territory and is marked by social contrasts among different regions and states, which showed contrasting data regarding the impact caused by COVID-19.
COVID-19 databases and data sharing are important to provide an overview of epidemiological aspects of the disease; however, Brazil lacks standardization in these datasets.

## 5. Limitations

Our study has limitations. First, the number of SARS-CoV-2 RT-PCR tests performed in Brazil is not fully clear, as there is limited access to these data in the official channels. Lack of official data availability is also an issue for number of new ICU beds and for the amount paid for mechanical ventilators and laboratory and hospital supplies. The disclosure of COVID-19 data was assigned to states and to the Federal District, which makes data normalization difficult. Also, many of our sources included preprints, which have not been peer-reviewed, and press articles, which, although not being scientific, were the only channels where some important and essential information for our review could be found.

## 6. Conclusions

After one year of COVID-19, Brazil certainly failed to manage the diseases spread. In the present article, we explored several factors associated with this failure, such as the low availability of SARS-CoV-2 RT-PCR tests, lack of efficient health policies, political instability (especially in the Ministry of Health), use of knowingly inefficient treatment approaches, lack of special attention for the most susceptible populations, lack of accessibility to health care facilities, high treatment costs, and, most important, the sabotaging attitudes by President Jair Messias Bolsonaro during the pandemic, which are not at all expected from an authority and to which an important part in the countrys current situation can be credited. Unfortunately, Brazil is maybe the best example of what not to do during a pandemic and indicates a need for a suitable government that can base its health policies on science and not personal political issues or guesswork.

## Additional Files

The additional files for this article can be found as follows:

10.5334/aogh.3182.s1 Supplement 1. Description of the Indigenous Health Special Districts in Brazil.

10.5334/aogh.3182.s2 Supplement 2. Number of Vaccinations dosages, SARS-CoV-2 RT-PCR, confirmed COVID-19 cases, deaths due COVID-19 and lethality for the data retrieved from 112 countries and territories.

## References

[1] Petersen E, Koopmans M, Go U, et al. Comparing SARS-CoV-2 with SARS-CoV and influenza pandemics. Lancet Infect Dis. 2020; 20: E238 E244. DOI: 10.1016/S1473-3099(20)30484-9 32628905 PMC7333991

[2] Ferguson N, Laydon D, Nedjati Gilani G, et al. Report 9: Impact of non-pharmaceutical interventions (NPIs) to reduce COVID19 mortality and healthcare demand. Imperial College COVID-19 Response Team. 2020. DOI: 10.25561/77482

[3] Liu Y, Yan LM, Wan L, et al. Viral dynamics in mild and severe cases of COVID-19. Lancet Infect Dis. 2020; 20(6): 656 657. DOI: 10.1016/S1473-3099(20)30232-2 32199493 PMC7158902

[4] Andrade RO. Covid-19 is causing the collapse of Brazils national health service. BMJ. 2020; 370: m3032. DOI: 10.1136/bmj.m3032 32732376

[5] Lefebvre M, Vignier N, Pitard B, et al. Covid-19 vaccines: Frequently asked questions and updated answers. Infect Dis Now. 2021. Epub Ahead of Print. DOI: 10.1016/j.idnow.2021.02.007 PMC7910656 33681861

[6] RECOVERY Collaborative Group, Horby P, Lim WS, et al. Dexamethasone in hospitalized patients with COVID-19. N Engl J Med. 2021; 384(8): 693 704. DOI: 10.1056/NEJMoa2021436 32678530 PMC7383595

[7] Polack FP, Thomas SJ, Kitchin N, et al. Safety and efficacy of the BNT162b2 mRNA Covid-19 vaccine. N Engl J Med. 2020; 383(27): 2603 2615. DOI: 10.1056/NEJMoa2034577 33301246 PMC7745181

[8] World Health Organization. 2020. Accessed on 12 March 2021. Available at https://www.who.int/

[9] Ministrio da Sade (Ministry of Health). Accessed on 12 March 2021. Available at https://www.gov.br/saude/pt-br

[10] WorldOMeter. Cases of Coronavirus in Brazil. 2020. Accessed on 12 March 2021. Available at https://www.worldometers.info/coronavirus/country/brazil/.

[11] Hallal PC, Horta BL, Barros AJD, et al. Evoluo da prevalncia de infeco por COVID-19 no Rio Grande do Sul, Brasil: inquritos sorolgicos seriados. Cien Saude Colet. 2020; 25(1): 2395 2401. DOI: 10.1590/1413-81232020256.1.09632020 32520284

[12] Fundao Oswaldo Cruz (Oswaldo Cruz Foundation). Monitoramento de casos reportados de sndrome respiratria aguda grave (SRAG) hospitalizados (Monitoring of reported cases of severe acute respiratory syndrome (SARS) hospitalized). Accessed on 12 March 2021. Available at http://info.gripe.fiocruz.br/.

[13] The White House. National Strategy for the COVID-19 Response and Pandemic Preparedness. 2021. Accessed on 12 March 2021. Available at https://www.whitehouse.gov/wp-content/uploads/2021/01/National-Strategy-for-the-COVID-19-Response-and-Pandemic-Preparedness.pdf

[14] Carvalho TA, Boschiero MN, Marson FAL. COVID-19 in Brazil: 150,000 deaths and the Brazilian underreporting. Diagn Microbiol Infect Dis. 2021; 99(3): 115258. DOI: 10.1016/j.diagmicrobio.2020.115258 33279819 PMC7576323

[15] Raoofi A, Takian A, Akbari Sari A, Olyaeemanesh A, Haghighi H, Aarabi M. COVID-19 pandemic and comparative health policy learning in Iran. Arch Iran Med. 2020; 23(4): 220 234. DOI: 10.34172/aim.2020.02 32271594

[16] Javed B, Sarwer A, Soto EB, Mashwani ZU. Is Pakistans response to Coronavirus (SARS-CoV-2) Adequate to prevent an outbreak? Front Med (Lausanne). 2020; 7: 158. DOI: 10.3389/fmed.2020.00158 32373620 PMC7187779

[17] Algaissi AA, Alharbi NK, Hassanain M, Hashem AM. Preparedness and response to COVID-19 in Saudi Arabia: Building on MERS experience. J Infect Public Health. 2020; 13(6): 834 838. DOI: 10.1016/j.jiph.2020.04.016 32451260 PMC7211706

[18] Edquist K, Jimnez MM. Spains policy response to the novel coronavirus and COVID-19. Research Gate. 2020.

[19] Anwar S, Nasrullah M, Hosen MJ. COVID-19 and Bangladesh: Challenges and how to address them. Front Public Health. 2020; 8: 154. DOI: 10.3389/fpubh.2020.00154 32426318 PMC7203732

[20] Djalante R, Lassa J, Setiamarga D, et al. Review and analysis of current responses to COVID-19 in Indonesia: Period of January to March 2020. Progress in Disaster Science. 2020; 6: 100091. DOI: 10.1016/j.pdisas.2020.100091 PMC7149002 34171011

[21] AlTakarli NS. Chinas response to the COVID-19 outbreak: A model for epidemic preparedness and management. Dubai Med J. 2020. DOI: 10.1159/000508448

[22] Vallejo BM, Jr, Ong RAC. Policy responses and government science advice for the COVID 19 pandemic in the Philippines: January to April 2020. Progress in Disaster Science. 2020; 7: 100115. Epub ahead of print. DOI: 10.1016/j.pdisas.2020.100115 PMC7299863 34173440

[23] Hale W, Petherick P, Kira. Oxford COVID-19 Government Response Tracker; 2020. DOI: 10.1038/s41562-021-01079-8 33686204

[24] Our World in Data. Accessed on 12 March 2021. Available at https://ourworldindata.org/policy-responses-covid3.

[25] Moreira RS. COVID-19: Intensive care units, mechanical ventilators, and latent mortality profiles associated with case-fatality in Brazil. Cad Sade Pblica. 2020; 36(5): e00080020. DOI: 10.1590/0102-311x00080020 32428072

[26] Imai N, Gaythorpe KAM, Abbott S, et al. Adoption and impact of non-pharmaceutical interventions for COVID-19. Wellcome Open Res. 2020; 5: 59. DOI: 10.12688/wellcomeopenres.15808.1 32529040 PMC7255913

[27] Moris D, Schizas D. Lockdown during COVID-19: The Greek success. In Vivo. 2020; 34(3): 1695 1699. DOI: 10.21873/invivo.11963 32503831 PMC8378029

[28] Taghrir MH, Akbarialiabad H, Ahmadi Marzaleh M. Efficacy of mass quarantine as leverage of health system governance during COVID-19 outbreak: A mini policy review. Arch Iran Med. 2020; 23(4): 265 267. DOI: 10.34172/aim.2020.08 32271600

[29] Sud SR. COVID-19 and keeping clean: A narrative review to ascertain the efficacy of personal protective equipment to safeguard health care workers against SARS-CoV-2. Hosp Pediatr. 2020; 10(7): 570 576. DOI: 10.1542/hpeds.2020-0135 32385055

[30] Tirupathi R, Bharathidasan K, Palabindala V, Salim SA, Al-Tawfiq JA. Comprehensive review of mask utility and challenges during the COVID-19 pandemic. Infez Med. 2020; 28(1): 57 63. 32532940

[31] Rawson T, Brewer T, Veltcheva D, Huntingford C, Bonsall MB. How and when to end the COVID-19 lockdown: an optimization approach. Front Public Health. 2020; 8: 262. DOI: 10.3389/fpubh.2020.00262 32587844 PMC7298102

[32] Federal Government of Brazil (Governo Federal do Brasil). Governo Federal j entregou mais de 8,4 mil ventiladores pulmonares (Federal Government has already delivered more than 8,400 pulmonary ventilators). Accessed on 12 March 2021. Available at https://www.gov.br/pt-br/noticias/saude-e-vigilancia-sanitaria/2020/07/governo-federal-ja-entregou-mais-de-8-4-mil-ventiladores-pulmonares.

[33] Revista Exame. Sistema de sade do Amazonas entra em colapso com a pandemia de coronavirus (Amazon health system collapses with coronavirus pandemic). 2020. Accessed on 17 March 2021. Available at https://exame.com/brasil/sistema-de-saude-do-amazonas-entra-em-colapso-com-pandemia-de-coronavirus/.

[34] Leal DFDVB, Santana da Silva MN, Fernandes DCRO, et al. Amerindian genetic ancestry as a risk factor for tuberculosis in an Amazonian population. PLoS One. 2020; 15(7): e0236033. DOI: 10.1371/journal.pone.0236033 32673332 PMC7365596

[35] Ellwanger JH, Kulmann-Leal B, Kaminski VL, et al. Beyond diversity loss and climate change: Impacts of Amazon deforestation on infectious diseases and public health. An Acad Bras Cienc. 2020; 92(1): e20191375. DOI: 10.1590/0001-3765202020191375 32321030

[36] Herrera D, Pfaff A, Robalino J. Impacts of protected areas vary with the level of government: Comparing avoided deforestation across agencies in the Brazilian Amazon. Proc Natl Acad Sci USA. 2019; 116(30): 14916 14925. DOI: 10.1073/pnas.1802877116 31285315 PMC6660721

[37] Baqui P, Bica I, Marra V, Ercole A, van der Schaar M. Ethnic and regional variations in hospital mortality from COVID-19 in Brazil: A cross-sectional observational study. Lancet Glob Health. 2020; 8(8): e1018 e1026. DOI: 10.1016/S2214-109X(20)30285-0 32622400 PMC7332269

[38] Marson F. Um milho de casos de COVID-19. Rev Med. 2020; 99(2): 209 212. DOI: 10.11606/issn.1679-9836.v99i2p209-212

[39] Instituto Brasileiro de Geografia e Estatstica (Brazilian Institute of Geography and Statistics). 2018. Accessed on 17 March 2021. Available at https://www.ibge.gov.br/ IBGE, 2018.

[40] Agncia Nacional de Sade Suplementar (National Supplementary Health Agency). 2020. Accessed on 12 March 2021. Available at http://www.ans.gov.br/planos-de-saude-e-operadoras/espaco-da-operadora/18-planos-de-saude-e-operadoras/espaco-da-operadora/263-ressarcimento-ao-sus.

[41] Associao de Medicina Intensiva Brasileira (Brazilian Intensive Care Association). AMIB apresenta dados atualizados sobre leitos de UTI no Brasil (AMIB shows updated data regarding ICU beds in Brazil). 2020. Accessed on 15 March 2021. Available at https://www.amib.org.br/fileadmin/user_upload/amib/2020/abril/28/dados_uti_amib.pdf.

[42] Registro Nacional de Terapia Intensiva (Intensive Care National Registry). UTIs Brasileiras (Brazilian ICU beds). 2021. Accessed on 10 March 2021. Available at http://www.utisbrasileiras.com.br/sari-covid-19/benchmarking-covid-19/.

[43] Publica, agncia de jornalismo investigativo (Public, Investigative Journalism Agency). 2020. Accessed on 12 March 2021. Available at: https://apublica.org/2020/05/enquanto-leitos-de-uti-do-sus-chegam-ao-limite-ha-vagas-nos-hospitais-privados/.

[44] COVID-19 Health Systems Response Monitor (HSRM). 2020. Accessed on 12 March 2021. Available at: http://covid19healthsystem.org.

[45] Senado Federal (Federal Senate). Projeto de Lei n2308 de 2020 (Law Project n2308 from 2020). 2020. Accessed on 10 March 2021. Available at https://www25.senado.leg.br/web/atividade/materias/-/materia/141752#:~:text=Ementa%3A,19%2C%20e%20d%C3%A1%20outras%20provid%C3%AAncias.

[46] Estado, Jornal (Estado Journal). Pblico e Privado versus COVID-19 (Public and Private versus COVID-19). 2020. Accessed on 12 March 2021. Available at: https://politica.estadao.com.br/blogs/fausto-macedo/publico-e-privado-versus-covid-19/.

[47] Folha de So Paulo, Jornal (Folha de So Paulo, Journal). As falhas evidenciadas pela COVID-19 no sistema privado de sade (The flaws in the private health system, evidenced by COVID-19). 2020. Accessed on 12 March 2021. Available at https://www1.folha.uol.com.br/opiniao/2020/04/as-falhas-evidenciadas-pela-covid-19-no-sistema-privado-de-saude.shtml.

[48] Folha de So Paulo, Jornal (Folha de So Paulo, Journal). Prefeitura investiga prevent senior por no informar casos de coronavirus (City hall investigate prevent senior for not informing cornavirus cases) 2020. Accessed on 12 March 2021. Available at https://www1.folha.uol.com.br/colunas/monicabergamo/2020/03/prefeitura-investiga-prevent-senior-por-nao-informar-casos-de-coronavirus.shtml.

[49] de Souza WM, Buss LF, Candido DDS, et al. Epidemiological and clinical characteristics of the COVID-19 epidemic in Brazil. Nat Hum Behav. 2020. Epub ahead of print. DOI: 10.1038/s41562-020-0928-4 32737472

[50] Palamim CVC, Marson FAL. Coronavirus disease: 4 million cases worldwide and the importance of multidisciplinary health care teams during the pandemic. J Emerg Nurs. 2020: S00991767(20)30202 6. DOI: 10.1016/j.jen.2020.06.004 PMC7284258 32605721

[51] Oliveira BA, Oliveira LC, Sabino EC, Okay TS. SARS-CoV-2 and the COVID-19 disease: A mini review on diagnostic methods. Rev Inst Med Trop S Paulo. 2020; 62. DOI: 10.1590/s1678-9946202062044 PMC7325591 32609256

[52] Sheridan C. Fast, portable tests come online to curb coronavirus pandemic. Nat Biotechnol. 2020; 38(5): 515 518. DOI: 10.1038/d41587-020-00010-2 32203294

[53] Jin Y, Wang M, Zuo Z, et al. Diagnostic value and dynamic variance of serum antibody in coronavirus disease 2019. Int J Infect Dis. 2020; 94: 49 52. DOI: 10.1016/j.ijid.2020.03.065 32251798 PMC7194885

[54] Okba NMA, Mller MA, Li W, et al. Severe Acute Respiratory Syndrome Coronavirus 2-specific antibody responses in coronavirus disease patients. Emerg Infect Dis. 2020; 26(7): 1478 1488. DOI: 10.3201/eid2607.200841 32267220 PMC7323511

[55] Lisboa Bastos M, Tavaziva G, Abidi SK, et al. Diagnostic accuracy of serological tests for covid-19: Systematic review and meta-analysis. BMJ. 2020; 370: m2516. DOI: 10.1136/bmj.m2516 32611558 PMC7327913

[56] Marson FAL. COVID-19 Six million cases worldwide and an overview of the diagnosis in Brazil: A tragedy to be announced. Diagn Microbiol Infect Dis. 2020; 98: 115113. DOI: 10.1016/j.diagmicrobio.2020.115113 32682217 PMC7834367

[57] The Guardian. Mass testing is the only way to stop the virus Its long overdue. Accessed on 13 March 2021. Available at https://www.theguardian.com/commentisfree/2020/mar/25/mass-covid-19-testing-is-vital-but-the-data-must-be-localised.

[58] Notcias UOL (UOL news). Novo teste de Bolsonaro para coronavrus tem resultado positivo (Bolsonaro has a new positive result for coronavirus). Accessed on 12 March 2021. Available at https://noticias.uol.com.br/politica/ultimas-noticias/2020/07/22/bolsonaro-teste-coronavirus.htm

[59] Notcias UOL (UOL news). Av de Michelle Bolsonaro morre por complicaes da covid-19 (Granmother of Michelle Bolsonaro dies due to COVID-19 complications). Notcias UOL. Av de Michelle Bolsonaro morre por complicaes da covid-19. Accessed on 12 March 2021. Available at https://noticias.uol.com.br/politica/ultimas-noticias/2020/08/12/avo-de-michelle-bolsonaro-morre-por-complicacoes-da-covid-19.htm.

[60] Portal Terra. Sexto ministro do governo Bolsonaro testa positivo para covid-19 (Sixth minister from the Bolsonaro government tests positive for COVID-19). Accessed on 12 March 2021. Available at https://www.terra.com.br/noticias/brasil/sexto-ministro-do-governo-bolsonaro-testa-positivo-para-covid-19,638e3eb2c278146d0e701739a05aed95i417foy1.html.

[61] Marson FAL, Ortega MM. COVID-19 in Brazil. Pulmonology. 2020; 26(4): 241 244. DOI: 10.1016/j.pulmoe.2020.04.008 32371054 PMC7183991

[62] Notcias UOL (UOL news). Bolsonaro diz que mscara tem eficcia quase nula; cincia aponta proteo (Bolsonaro says facial masks have almost zero efficiency; science points protection). Accessed on 12 March 2021. Available at https://noticias.uol.com.br/saude/ultimas-noticias/redacao/2020/08/19/bolsonaro-mascara-eficacia.htm.

[63] Notcias UOL (UOL news). Bolsonaro passeia e encontra seguidores aps afirmar no ter mais covid-19 (Bolsonaro strolls and meets followers after afirming he no longer has COVID-19). Accessed on 12 March 12, 2021. Available at https://noticias.uol.com.br/politica/ultimas-noticias/2020/07/25/bolsonaro-deixa-planalto-e-passeia-de-moto-apos-teste-negativo-para-covid.htm.

[64] Hartley K, Vu MK. Fighting fake news in the COVID-19 era: Policy insights from an equilibrium model. Policy Sci. 2020:1 24. Epub ahead of print. DOI: 10.1007/s11077-020-09405-z PMC7479406 32921821

[65] Hallal PC. SOS Brazil: Science under attack. Lancet. 2021; 397(10272): 373 374. DOI: 10.1016/S0140-6736(21)00141-0 PMC7825897 33493436

[66] Silva DN. The pragmatics of chaos: Parsing Bolsonaros undemocratic language. Trab Linguist Apl. 2020; 59(1): 507 537. DOI: 10.1590/01031813685291420200409

[67] Garret L. COVID-19: The medium is the message. Lancet. 2020; 395: 942 943. DOI: 10.1016/S0140-6736(20)30600-0 32171075 PMC7154501

[68] The Lancet. COVID-19 in Brazil: So what? Lancet. 2020; 395: 1461. DOI: 10.1016/S0140-6736(20)31095-3 32386576 PMC7251993

[69] Social isolation So Paulo State. Accessed on 12 March 2021. Available at https://www.saopaulo.sp.gov.br/coronavirus/isolamento/.

[70] Silva RDFC, Gonalves LAP. As plulas do Messias: Salvao, negao e poltica de morte em tempos de pandemia. Physis. 2020; 30(2): e300208. DOI: 10.1590/s0103-73312020300208

[71] Paumgartten FJR. Sobre a alegada eficcia anticncer da plula de fosfoetanolamina, fragilidade da evidncia cientfica e preocupaes ticas. Vigil Sanit Debate. 2016; 4(3): 4 12. DOI: 10.22239/2317-269x.00822pt

[72] Caetano NAPO, Moreira TC, Ugrinovich LA, Carmo TA, Simioni PU. A pesquisa com a fosfoetanolamina sinttica como inibidor da progresso de tumores. Rev Fac Cinc Md Sorocaba. 2017; 19(3): 111 116. DOI: 10.23925/1984-4840.2017v19i3a3

[73] Gautret P, Lagier JC, Parola P, et al. Hydroxychloroquine and azithromycin as a treatment of COVID-19: Results of an open-label non-randomized clinical trial. Int J Antimicrob Agents. 2020; 56(1): 105949. DOI: 10.1016/j.ijantimicag.2020.105949 32205204 PMC7102549

[74] Gautret P, Hoang VT, Honor S, et al. Response to the use of hydroxychloroquine in combination with azithromycin for patients with COVID-19 is not supported by recent literature. Int J Antimicrob Agents. 2021; 57(1): 106241. DOI: 10.1016/j.ijantimicag.2020.106241 33408031 PMC7779257

[75] Rosendaal FR. Review of: Hydroxychloroquine and azithromycin as a treatment of COVID-19: Results of an open-label non-randomized clinical trial Gautret et al 2010, Int J Antimicrob Agents. 2020; 56(1): 106063. DOI: 10.1016/j.ijantimicag.2020.106063 32674928 PMC7357515

[77] Poltica Estado (Policy Estado). Bolsonaro reitera uso de cloroquina e diz que indicaria medicamento para me (Bolsonaro reiterates the use of chloroquine and says he would indicate it to his mother). Accessed on 12 March 2021. Available at https://politica.estadao.com.br/noticias/geral,bolsonaro-reitera-uso-da-cloroquina-e-diz-que-indicaria-medicamento-para-a-mae,70003265327.

[78] The Scientist. Journal Publisher Concerned over Hydroxychloroquine Study. Accessed on 13 March 2021. Available at https://www.the-scientist.com/news-opinion/journal-publisher-concerned-over-hydroxychloroquine-study-67405.

[79] O Globo. Entidades especialistas em sade condenam manifesto que defende tratamento precoce da COVID-19 (Especialist entities condemn manifest on earlier treatment for COVID-19). 2020. Accessed on 12 March 2021. Available at https://oglobo.globo.com/sociedade/entidades-especialistas-em-saude-condenam-manifesto-que-defende-tratamento-precoce-da-covid-19-24895651.

[80] Develoux M. Ivermectine [Ivermectin]. Ann Dermatol Venereol. 2004; 131(67 Pt 1): 561 570. DOI: 10.1016/S0151-9638(04)93668-X 15318139

[81] Lpez-Medina E, Lpez P, Hurtado IC, et al. Effect of Ivermectin on time to resolution of symptoms among adults with mild COVID-19: A randomized clinical trial. JAMA. 2021. DOI: 10.1001/jama.2021.3071 PMC7934083 33662102

[82] MERCK. Merck Statement on Ivermectin use During the COVID-19 Pandemic. 2021. Accessed on 10 March 2021. Available at https://www.merck.com/news/merck-statement-on-ivermectin-use-during-the-covid-19-pandemic.

[83] Zou L, Dai L, Zhang X, Zhang Z, Zhang Z. Hydroxychloroquine and chloroquine: A potential and controversial treatment for COVID-19. Arch Pharm Res. 2020. Epub ahead of print. DOI: 10.1007/s12272-020-01258-7 PMC7395211 32740801

[84] Tang D, Li J, Zhang R, Kang R, Klionsky DJ. Chloroquine in fighting COVID-19: Good, bad, or both? Autophagy. 2020. Epub ahead of print. DOI: 10.1080/15548627.2020.1796014 PMC7751499 32713288

[85] Khuroo MS, Sofi AA, Khuroo M. Chloroquine and Hydroxychloroquine in Coronavirus Disease 2019 (COVID-19). Facts, Fiction & the Hype. A Critical Appraisal. Int J Antimicrob Agents. 2020: 106101. Epub ahead of print. DOI: 10.1016/j.ijantimicag.2020.106101 PMC7366996 32687949

[86] Cortegiani A, Ippolito M, Ingoglia G, Iozzo P, Giarratano A, Einav S. Update I. A systematic review on the efficacy and safety of chloroquine/hydroxychloroquine for COVID-19. J Crit Care. 2020; 59: 176 190. Epub ahead of print. DOI: 10.1016/j.jcrc.2020.06.019 32683212 PMC7351664

[87] Cavalcanti AB, Zampieri FG, Rosa RG, et al. Hydroxychloroquine with or without Azithromycin in Mild-to-Moderate Covid-19. N Engl J Med. 2020. Epub ahead of print. DOI: 10.1056/NEJMoa2019014 PMC7397242 32706953

[88] Chaccour C, Casellas A, Blanco-Di Matteo A, et al. The effect of early treatment with ivermectin on viral load, symptoms and humoral response in patients with non-severe COVID-19: A pilot, double-blind, placebo-controlled, randomized clinical trial. EClinicalMedicine. 2021; 32: 100720. DOI: 10.1016/j.eclinm.2020.100720 33495752 PMC7816625

[89] Galan LEB, Santos NMD, Asato MS, et al. Phase 2 randomized study on chloroquine, hydroxychloroquine or ivermectin in hospitalized patients with severe manifestations of SARS-CoV-2 infection. Pathog Glob Health. 2021. Epub ahead of print. DOI: 10.1080/20477724.2021.1890887 PMC7938655 33682640

[90] Folha de So Paulo, Jornal (Folha de So Paulo, Journal). Pazuello Ministry under investigation by federal police and at worst moment of the pandemic. 2021. Accessed on 16 March 2021. Available at https://www1.folha.uol.com.br/internacional/en/brazil/2021/03/pazuello-leaves-ministry-under-investigation-by-federal-police-and-at-worst-moment-of-the-pandemic.shtml.

[91] Notcias UOL (UOL News). Ministrio ignorou oferta de 160 milhes de doses de coronavac em julho, diz Butantan (Ministry ignored 160 million dosages of cornavac vaccine on July, says Butantan). 2021. Accessed on 16 March 2021. Available at https://noticias.uol.com.br/saude/ultimas-noticias/reuters/2021/02/19/ministerio-ignorou-oferta-de-160-milhoes-de-doses-da-coronavac-em-julho-diz-butantan.htm.

[92] He D, Artzy-Randrup Y, Musa SS, Stone L. The unexpected dynamics of COVID-19 in Manaus. Brazil: Herd immunity versus interventions. 2021. [Pre-Print]. *MedRxiv*. DOI: 10.1101/2021.02.18.21251809

[93] O Globo. Saiba quem Marcelo Queiroga indicadoa por Bolsonaro para ser novo ministro da sade (Find out who Marcelo Queiroga, is, indicated by Bolsonaro to be the next ministry of health). 2021. Accessed on 17 March 2021. Available at https://g1.globo.com/politica/noticia/2021/03/15/saiba-quem-e-marcelo-queiroga-indicado-por-bolsonaro-para-ser-o-novo-ministro-da-saude.ghtml.

[94] Reuters. Brazils incoming health minister says mission is to unify fight against COVID-19. 2021. Accessed on 17 March 2021. Available at https://www.reuters.com/article/health-coronavirus-brazil-minister-idINKBN2B92E8.

[95] Shang Y, Pan C, Yang X, et al. Management of critically ill patients with COVID-19 in ICU: Statement from front-line intensive care experts in Wuhan, China. Ann Intensive Care. 2020; 10: 73. DOI: 10.1186/s13613-020-00689-1 32506258 PMC7275657

[96] CNN Brasil (CNN Brazil). Preo de respirador comprado por estados varia at 4 vezes e enfrenta apurao (Price of ventilator purchased by states vary until 4 times and faces counting). Accessed on 12 March 2021. Available at https://www.cnnbrasil.com.br/saude/2020/05/26/preco-de-respirador-comprado-por-estados-varia-ate-4-vezes-e-enfrenta-apuracoes.

[97] The Intercept Brazil. Respiradores fantasmas (Ghost Ventilators). 2020. Accessed on March 12, 2021. Available at https://theintercept.com/series/respiradores/.

[98] Conselho Nacional de Secretrios da Sade (National Council of Health Secretaries). Accessed on March 12, 2021. Available at https://www.conass.org.br.

[99] Agncia Brasil (Brazil Agency). Relatrio aponta desabastecimento de remdio nos estados (Report shows shortage of drugs in states). Accessed on 12 March 2021. Available at https://agenciabrasil.ebc.com.br/saude/noticia/2020-06/covid-19-relatorio-aponta-desabastecimento-de-remedios-nos-estados.

[100] Sikkema RS, Pas SD, Nieuwenhuijse DF, et al. COVID-19 in health-care workers in three hospitals in the south of the Netherlands: A cross-sectional study. Lancet Infect Dis. 2020; 10: 1273 1280. DOI: 10.1016/S1473-3099(20)30527-2 PMC7332281 32622380

[101] Pan A, Liu L, Wang C, et al. Association of public health interventions with the epidemiology of the COVID-19 outbreak in Wuhan, China. JAMA. 2020; 323(19): 1915 1923. DOI: 10.1001/jama.2020.6130 32275295 PMC7149375

[102] Chou R, Dana T, Buckley DI, Selph S, Fu R, Totten AM. Epidemiology of and risk factors for coronavirus infection in health care workers: A living rapid review. Ann Intern Med. 173(2): 120 136. DOI: 10.7326/M20-1632 PMC7240841 32369541

[103] International Council of Nurses. International council of nurses COVID-19 update. 2021. Accessed on 15 March 2021. Available at https://www.icn.ch/sites/default/files/inline-files/ICN%20COVID19%20update%20report%20FINAL.pdf.

[104] Conselho Federal de Enfermagem (Brazilian Federal Nursery Council). Brasil representa um tero das mortes de profissionais de Enfermagem por covid-19 (Brazil represents one third of the nurses deaths due to COVID-19). 2021. Accessed on 15 March 2021. Available at http://www.cofen.gov.br/brasil-responde-por-um-terco-das-mortes-de-profissionais-de-enfermagem-por-covid-19_84357.html.

[105] Black JRM, Bailey C, Przewrocka J, Dijkstra KK, Swanton C. COVID-19: The case for health-care worker screening to prevent hospital transmission. Lancet. 2020; 395(10234): 1418 1420. DOI: 10.1016/S0140-6736(20)30917-X 32305073 PMC7162624

[106] Adams JG, Walls RM. Supporting the health care workforce during the COVID-19 global epidemic. JAMA. 2020; 323: 1439 1440. DOI: 10.1001/jama.2020.3972 32163102

[107] Perlis RH. Exercising heart and head in managing coronavirus disease 2019 in Wuhan. JAMA Netw Open. 2020; 3: e204006. DOI: 10.1001/jamanetworkopen.2020.4006 32202641

[108] BBC News. Coronavirus: Trump moves to pull US out of World Health Organization. Published on 7 7, 2020. Accessed on 12 March 2021. Available at https://www.bbc.com/news/world-us-canada-53327906#:~:text=President%20Donald%20Trump%20has%20formally,wake%20of%20the%20coronavirus%20pandemic.

[109] Blomm DE, Canning D. Population health and economic growth. World Bank Group-Health and Growth. 2009. Accessed on 15 March 2021. Available at https://openknowledge.worldbank.org/handle/10986/28036.

[110] Buss LF, Prete, CA, Jr, Abrahim CMM, et al. Three-quarters attack rate of SARS-CoV-2 in the Brazilian Amazon during a largely unmitigated epidemic. Science. 2021; 371(6526): 288 292. DOI: 10.1126/science.abe9728 33293339 PMC7857406

[111] Faria NR, Mellan TA, Whittaker C, et al. Genomics and epidemiology of a novel SARS-CoV-2 lineage in Manaus, Brazil. medRxix. DOI: 10.1101/2021.02.26.21252554 PMC8139423 33853970

[112] Murray J. Has Swedens controversial covid-19 strategy been successful or not? BMJ. 2020; 370: m3255. DOI: 10.1136/bmj.m3255 32843334

[113] Thanh Le T, Andreadakis Z, Kumar A, et al. The COVID-19 vaccine development landscape. Nat Rev Drug Discov. 2020; 19(5): 305 306. DOI: 10.1038/d41573-020-00073-5 32273591

[114] Domingues CMAS. Desafios para a realizao da campanha de vacinao contra a COVID-19 no Brasil. Cad Sade. 2021; 37(1): e00344620. DOI: 10.1590/0102-311x00344620 33440416

[115] Agncia Brasil. Govt earmarks $1.38 bi for purchase of Janssen and Pfizer vaccines. Accessed on 12 March 2021. Available at https://agenciabrasil.ebc.com.br/en/saude/noticia/2021-03/govt-earmarks-138-bi-purchase-janssen-and-pfizer-vaccines.

[116] Agncia Brasil. Brazil buys another 54 mi doses of vaccine against COVID-19. Accessed on 12 March 2021. Available at https://agenciabrasil.ebc.com.br/en/saude/noticia/2021-02/brazil-buys-another-54-mi-doses-vaccine-against-covid-19.

[117] Portal IG de Notcias. Ministrio da Sade promete 424,5 milhes de doses de vacina at fim do ano (Ministry of Health promises 424.5 million dosages of vaccine by the end of the year). Accessed on 12 March 2021. Available at https://saude.ig.com.br/coronavirus/2021-03-12/ministerio-da-saude-promete-4245-milhoes-de-doses-de-vacina-ate-fim-do-ano.html.

[118] CNN World. How Brazils Covid-19 vaccine plan fell apart. Accessed on 12 March 2021. Available at https://edition.cnn.com/2021/02/01/americas/brazil-coronavirus-vaccination/index.html.

[119] El Pais. Bolsonaro celebra como uma vitria a suspenso dos testes da Coronavac (Bolsonaro celebrates the suspension of Coronavac tests as a victory). Accessed on 12 March 2021. Available at https://brasil.elpais.com/brasil/2020-11-10/bolsonaro-celebra-como-uma-vitoria-a-suspensao-dos-testes-da-coronavac.html.

[120] Sato APS. What is the importance of vaccine hesitancy in the drop of vaccination coverage in Brazil? Ver Sade Pbl. 2018; 52: 96. DOI: 10.11606/S1518-8787.2018052001199 PMC6284490 30517523

[121] Governo do Estado de So Paulo. Governo de SP inicia em Serrana teste indito de vacinao em massa (SP government starts in Serrana unprecedented mass vaccination test). Accessed on 12 March 2021. Available at https://www.saopaulo.sp.gov.br/noticias-coronavirus/governo-de-sp-inicia-em-serrana-teste-inedito-de-vacinacao-em-massa-2/.

