# Supplementary material for: One Year of Coronavirus Disease 2019 (COVID-19) in Brazil: A Political and Social Overview
Source: Ann Glob Health. 2021 May 18;87(1):44. doi: 10.5334/aogh.3182 (PMC8139306; doi:10.5334/aogh.3182)
Supplement: Supplement 1. — Description of the Indigenous Health Special Districts in Brazil. [file agh-87-1-3182-s1.pdf]

Supplement 1. Description of the Indigenous Health Special Districts in Brazil.

DSEI	Base Municipality (FU)	General indigenous population assisted	Ethnic group	Tribe	CASAI	States covered	Municipalities covered	Base Center
Alagoas Sergipe	Maceió (AL)	12,250	12	31	0	Alagoas and Sergipe	10	ND
Altamira	Altamira (PA)	3,974	10	60	1	Pará	5	1
Alto Rio Negro	São Gabriel da Cachoeira (AM)	40,233	25	707	1	Amazonas	3	25
Alto Rio Juruá	Cruzeiro do Sul (AC)	17,672	17	148	1	Acre	8	7
Alto Rio Purus	Rio Branco (AC)	13,555	7	150	1	Amazonas, Acre and Rondônia	7	6
Alto Rio Solimões	Tabatinga (AM)	70,519	7	234	1	Amazonas	7	12
Alto Amapá and northern Pará	Macapá (AP)	12,440	11	139	2	Amapá and Pará	4	6
Araguaia	São Félix do Araguaia (MT)	5,562	8	41	1	Mato grosso, Goiás and Tocantins	12	4
Bahia	Salvador (BA)	29,284	21	77	0	Bahia	23	9
Ceará	Fortaleza (CE)	26,129	15	100	1	Ceará	16	9
Cuiabá	Cuiabá (MT)	6,830	10	120	3	Mato grosso	16	3
Guamá-Tocantins	Belém (PA)	13,913	138	153	5	Tocantins and Pará	17	8
Interior Sul	Florianópolis (SC)	63,118	4	180	0	São Paulo, Santa Catarina and Rio Grande do Sul	65	8
Kaiapó MT	Colíder (MT)	6,424	4	51	3	Pará and Mato Grosso	6	3
Kaiapó do Pará	Redenção (PA)	5,796	1	50	4	Pará	6	4
Leste Roraima	Boa Vista (RR)	53,213	7	323	0	Roraima	10	34
Litoral Sul	Curitiba (PR)	22,975	11	129	2	São Paulo, Santa Catarina, Rio Grande do Sul, Rio de Janeiro and Paraná	68	15
Manaus	Manaus (AM)	30,768	35	218	1	Amazonas	15	16
Maranhão	São Luís (MA)	36,060	8	424	3	Maranhão	16	6
Mato Grosso do Sul	Campo Grande (MS)	83,434	8	99	3	Mato Grosso do Sul	129	15
Médio Rio Purus	Lábrea (AM)	6,822	17	105	2	Amazonas	3	10
Médio Rio Solimões and affluents	Tefé (AM)	24,538	16	184	2	Amazonas	14	15
Minas Gerais and Espírito Santo	Governador Valadares (MG)	16,648	10	93	2	Minas Gerais and Espírito Santo	14	18
Parintins	Parintins (AM)	16,911	2	124	2	Amazonas and Pará	5	13
Pernambuco	Recife (PE)	39,231	13	224	1	Pernambuco	15	12
Porto Velho	Porto Velho (RO)	10,311	68	172	6	Rondônia, Mato Grosso and Amazonas	15	5
Pontiguara	João Pessoa (PB)	14,024	1	33	0	Paraíba	3	3
Rio Tapajós.	Itaituba (PA)	12,722	4	141	4	Pará	4	11
Tocantins	Palmas (TO)	11,908	11	160	2	Tocantins and Goiás	12	5
Vale do Javari	Atalaia do Norte (AM)	6,263	6	59	1	Amazonas	1	8
Vilhena	Cacoal (RO)	7,159	17	172	4	Amazonas	2	4
Xavante	Barra do Garças (MT)	20,653	1	305	1	Mato Grosso	12	6

Xingu	Canarana (MT)	7,213	16	81	4	Mato Grosso	8	4
Yanomami	Boa Vista (RR)	25,486	2	323	1	Roraima and Amazonas	1	37

ND, nothing declared; CASAI, Indigenous health houses; DSEI, Indigenous Health Special Districts; FU, federation unit and federal district (FD).
