# Supplementary material for: One Year of Coronavirus Disease 2019 (COVID-19) in Brazil: A Political and Social Overview
Source: Ann Glob Health. 2021 May 18;87(1):44. doi: 10.5334/aogh.3182 (PMC8139306; doi:10.5334/aogh.3182)
Supplement: Supplement 2. — Number of Vaccinations dosages, SARS-CoV-2 RT-PCR, confirmed COVID-19 cases, deaths due COVID-19 and lethality for the data retrieved from 112 countries and territories. [file agh-87-1-3182-s2.pdf]

Supplement 2. Number of Vaccination's dosages, SARS-CoV-2 RT-PCR, confirmed COVID-19 cases, deaths due COVID-19 and lethality for the data retrieved from 112 countries and territories.

Country	Total Vaccination (M)	Total Vaccination per 100	Total population	Confirmed Cases	Deaths	Lethality	Confirmed cases/1M	Death/1M	Recovered cases	Active cases	Number of tests	Number of tests/1M
World	335,440,000	4.30	7,851,741,930	119,580,941	2,650,476	2.22	15,229.86	337.57	96,233,327	20,697,138		
USA	98,200,000	29.40	333,346,783	29,988,140	545,324	1.82	89,960.79	1,635.91	22,027,776	7,415,040	376,068,767	1,131,555
Chi	52,520,000	3.60	1,439,323,776	90,027	4,636	5.15	62.55	3.22	85,209	182	160,000,000	111,163
India	26,160,000	1.90	1,389,382,742	11,333,484	158,483	1.40	8,157.21	114.07	10,971,329	203,672	224,998,638	161,941
United Kingdom	20,110,000	36.00	68,133,028	4,248,286	125,343	2.95	62,352.81	1,839.68	3,424,092	698,851	103,053,938	1,512,540
Brazil	10,740,000	5.00	213,605,167	11,368,317	276,276	2.43	53,221.17	1,293.40	10,000,981	1,092,061	28,600,001	134,892
Turkey	10,500,000	12.40	84,966,101	2,850,930	29,356	1.03	33,553.73	345.50	2,670,273	151,301	34,843,480	410,087
Israel	9,180,000	106.00	9,197,590	816,198	5,980	0.73	88,740.42	650.17	775,896	34,322	12,857,383	1,397,908
Germany	8,720,000	10.40	83,969,683	2,559,296	73,790	2.88	30,478.81	878.77	2,345,600	139,906	46,319,641	551,623
Russia	7,270,000	5.00	145,977,899	4,370,617	91,220	2.09	29,940.27	624.89	3,973,029	306,368	114,400,000	783,680
UnitedArab Emirates	6,410,000	64.80	9,972,913	422,246	1,378	0.33	42,339.28	138.17	401,539	19,329	33,348,059	3,343,863
Italy	6,220,000	10.30	60,400,064	3,175,807	101,564	3.20	52,579.53	1,681.52	2,564,926	509,317	43,976,394	728,085
Chile	6,130,000	32.10	19,229,693	879,485	21,451	2.44	45,735.78	1,115.51	824,899	33,135	9,982,597	519,124
Spain	5,170,000	11.10	46,767,349	3,183,704	72,258	2.27	68,075.36	1,545.05	2,857,714	253,732	40,292,390	861,550
Morocco	5,100,000	13.80	37,212,677	488,181	8,716	1.79	13,118.67	234.22	474,938	4,527	5,781,254	155,357
Indonesia	4,990,000	1.80	275,522,178	1,410,134	38,229	2.71	5,118.04	138.75	1,231,454	140,451	11,502,310	41,747
Poland	4,390,000	11.60	37,817,820	1,868,297	46,724	2.50	49,402.56	1,235.50	1,530,484	291,089	10,535,957	278,598
Bangladesh	4,010,000	2.40	165,823,454	555,222	8,515	1.53	3,348.27	51.35	509,172	37,535	4,232,139	25,522
Mexico	3,800,000	3.00	129,868,807	2,151,028	193,142	8.98	16,563.08	1,487.21	1,691,093	266,793	5,711,049	43,976
Cada	2,720,000	7.20	37,971,008	901,984	22,399	2.48	23,754.54	589.90	849,056	30,529	25,542,543	672,685
Romania	1,970,000	10.20	19,148,352	850,362	21,360	2.51	44,409.15	1,115.50	777,715	51,287	6,687,240	349,233
Argenti	1,920,000	4.30	45,482,534	2,185,747	53,578	2.45	48,056.84	1,177.99	1,974,866	157,303	7,929,640	174,345
Saudi Arabia	1,840,000	5.30	35,187,590	381,708	6,556	1.72	10,847.80	186.32	372,217	2,935	14,227,459	404,332
Serbia	1,830,000	26.90	8,712,896	507,959	4,670	0.92	58,299.67	535.99	400,347	102,942	3,122,905	358,423
Netherlands	1,620,000	9.40	17,161,169	1,144,822	16,024	1.40	66,710.02	933.74			6,970,400	406,173
Hungary	1,580,000	16.40	9,643,303	498,183	16,627	3.34	51,661.03	1,724.20	346,904	134,652	3,992,790	414,048
Greece	1,220,000	11.70	10,387,636	217,018	6,986	3.22	20,891.95	672.53	182,945	27,087	5,787,456	557,149
Portugal	1,080,000	10.60	10,176,043	813,152	16,650	2.05	79,908.47	1,636.20	749,77	46,732	8,480,932	833,421
Sweden	1,050,000	10.40	10,142,867	712,527	13,146	1.84	70,249.07	1,296.08			6,378,529	628,868
Belgium	1,040,000	9.00	11,624,575	798,108	22,370	2.80	68,656.96	1,924.37	54,044	721,694	9,954,558	856,337
Czechia	999,365	9.30	10,722,763	1,376,998	22,865	1.66	128,418.21	2,132.38	1,191,782	162,351	9,390,615	875,764
Switzerland	948,678	11.00	8,698,423	570,645	10,104	1.77	65,603.27	1,161.59	528,104	32,437	5,387,481	619,363

Austria	929,497	10.30	9,041,987	488,042	8,831	1.81	53,975.08	976.67	453,078	26,133	5,936,541	656,553
Denmark	796,630	13.80	5,806,342	219,305	2,387	1.09	37,769.91	411.10	208,601	8,317	19,996,218	3,443,858
Seri Lanka	752,098	3.50	21,475,470	87,286	525	0.60	4,064.45	24.45	83,958	2,803	2,225,675	103,638
Norway	659,720	12.20	5,450,570	78,946	639	0.81	14,483.99	117.24	66,014	12,293	3,910,562	717,459
Finland	625,039	11.30	5,546,692	65,315	786	1.20	11,775.49	141.71	46	18,529	3,572,800	644,132
Singapore	611,314	10.40	5,882,175	60,080	29	0.05	10,213.91	4.93	59,95	101	7,805,264	1,326,935
Dominican Republic	606,006	5.60	10,923,097	245,452	3,213	1.31	22,470.92	294.15	200,922	41,317	1,244,942	113,973
Slovakia	596,924	10.90	5,461,478	333,872	8,346	2.50	61,132.17	1,528.16	255,300	70,226	2,175,955	398,419
South Korea	546,924	1.10	51,299,807	94,686	1,662	1.76	1,845.74	32.40	85,743	7,281	7,012,664	136,700
Ireland	536,617	10.90	4,975,888	225,820	4,518	2.00	45,382.85	907.98	23,364	197,938	3,688,558	741,286
Bahrain	520,429	30.60	1,741,936	129,825	480	0.37	74,529.14	275.56	123,170	6,175	3,275,677	1,880,481
Colombia	480,250	0.90	51,257,626	2,294,617	60,950	2.66	44,766.35	1,189.09	2,195,569	38,098	11,918,588	232,523
Peru	440,829	1.30	33,288,708	1,394,571	48,484	3.48	41,893.21	1,456.47	1,301,439	44,648	8,105,930	243,504
Azerbayan	436,849	4.30	10,202,372	238,959	3,268	1.37	23,421.91	320.32		230,461	2,690,711	263,734
Nepal	402,264	1.40	29,497,597	275,118	3,012	1.09	9,326.79	102.11	271,177	929	2,210,434	74,936
Kuwait	360,000	8.40	4,314,122	207,249	1,156	0.56	48,039.67	267.96	191,417	14,676	1,893,722	438,959
Lithuania	333,292	12.20	2,695,772	204,356	3,373	1.65	75,806.11	1,251.22	190,234	10,749	2,202,876	817,160
Qatar	327,000	11.30	2,807,805	169,284	265	0.16	60,290.51	94.38	157,577	11,442	1,610,526	573,589
Bulgaria	324,446	4.70	6,912,019	275,859	11,196	4.06	39,910.05	1,619.79	223,48	41,183	1,826,934	264,313
Gha	300,000	1.00	31,518,821	86,737	656	0.76	2,751.91	20.81	81,299	4,782	929,189	29,480
Croatia	265,223	6.50	4,087,646	249,661	5,647	2.26	61,076.96	1,381.48	239,492	4,522	1,416,328	346,490
Rwanda	230,000	1.80	13,172,483	20,057	275	1.37	1,522.64	20.88	18,361	1,421	1,056,416	80,199
Pama	224,765	5.20	4,361,582	346,775	5,972	1.72	79,506.70	1,369.23	334,256	6,547	1,995,370	457,488
Malaysia	223,923	0.70	32,652,231	320,939	1,203	0.37	9,829.01	36.84	302,662	17,074	6,747,513	206,648
Slovenia	223,703	10.90	2,079,126	199,004	3,926	1.97	95,715.22	1,888.29	184,753	10,325	967,123	465,156
Costa Rica	204,586	4.00	5,126,283	209,093	2,862	1.37	40,788.42	558.30	188,967	17,264	729,069	142,222
Maldivas	186,930	34.60	547,100	21,382	64	0.30	39,082.43	116.98	18,780	2,538	568,887	1,039,823
Japan	181,184	0.10	126,207,313	444,289	8,451	1.90	3,520.31	66.96	423,619	12,219	8,834,799	70,002
Uruguay	170,134	4.90	3,482,076	69,074	689	1.00	19,837.02	197.87	58,627	9,758	1,114,405	320,040
Estonia	161,693	12.20	1,327,154	82,286	695	0.84	62,001.85	523.68	58,043	23,548	1,019,342	768,066
Jordan	150,000	1.50	10,273,146	464,856	5,224	1.12	45,249.62	508.51	385,533	74,099	5,103,809	496,811
Hong Kong	145,800	1.90	7,539,057	11,211	203	1.81	1,487.06	26.93	10,710	298	9,296,529	1,233,116
South Africa	138,014	0.20	59,824,880	1,526,873	51,179	3.35	25,522.37	855.48	1,449,654	26,040	9,393,727	157,020
Bolivia	132,286	1.10	11,782,882	257,240	11,903	4.63	21,831.67	1,010.19	202,216	43,121	783,751	66,516
Cambodia	129,908	0.80	16,878,593	1,225	1	0.08	72.58	0.06	619	605	555,192	32,893

Australia	125,000	0.50	25,704,223	29,102	909	3.12	1,132.19	35.36	26,222	1,971	14,833,456	577,082
Philippines	114,500	0.10	110,587,545	611,618	12,694	2.08	5,530.62	114.79	546,912	52,012	9,183,552	83,043
Malta	108,971	24.70	442,358	25,969	346	1.33	58,705.84	782.17	22,435	3,188	746,422	1,687,371
Cyprus	100,000	11.40	1,213,442	38,496	236	0.61	31,724.63	194.49	2,057	36,203	2,420,724	1,994,924
Oman	98,168	1.90	5,195,694	145,257	1,600	1.10	27,957.19	307.95	135,227	8,430	1,550,000	298,324
Lebanon	91,967	1.40	6,804,185	411,839	5,278	1.28	60,527.31	775.70	321,990	84,571	3,240,837	476,301
Latvia	91,086	4.80	1,871,656	92,906	1,746	1.88	49,638.40	932.86	83,382	7,778	1,652,688	883,008
Seychelles	87,333	88.80	98,769	3,173	16	0.50	32,125.46	161.99	2,847	310	5,200	52,648
Ecuador	72,258	0.40	17,827,757	299,216	16,193	5.41	16,783.72	908.30	256,009	27,014	1,059,747	59,444
Algeria	75,000	0.20	44,396,169	115,008	3,031	2.64	2,590.49	68.27	79,672	32,305		
Senegal	68,205	0.40	17,046,383	36,569	951	2.60	2,145.26	55.79	31,977	3,641	409,588	24,028
Luxembourg	51,457	8.20	632,995	57,503	681	1.18	90,842.74	1,075.84	53,941	2,881	2,228,285	3,520,225
Barbados	49,757	17.30	287,619	3,391	37	1.09	11,789.90	128.64	3,106	248	132,604	461,040
Iceland	46,052	13.50	342,776	6,072	29	0.48	17,714.19	84.60	6,023	20	513,439	1,497,885
Gilbratar	44,894	133.30	33,684	4,263	93	2.18	126,558.60	2,760.95	4,140	30	197,626	5,867,059
Jersey	39,428	39.00	174,971	4,043	86	2.13	23,106.69	491.51	3,955	2	388,235	2,218,853
Zimbabwe	36,019	0.20	15,012,137	36,423	1,496	4.11	2,426.24	99.65	33,996	931	355,865	23,705
Isle of Man	28,431	33.40	85,345	1,157	26	2.25	13,556.74	304.65	424	707	38,147	446,974
Cayman Islands	26,580	40.40	66,255	468	2	0.43	7,063.62	30.19	430	36	76,896	1,160,607
Bermuda	23,939	38.40	62,118	732	12	1.64	11,784.02	193.18	692	28	180,543	180,543
Kazakhstan	22,294	0.10	18,931,796	221,669	2,837	1.28	11,708.82	149.85	204,782	14,050	7,925,003	418,608
Albania	21,613	0.80	2,875,614	116,123	2,018	1.74	40,381.98	701.76	79,131	34,974	500,094	173,909
Belarus	20,944	0.20	9,447,165	300,146	2,078	0.69	31,771.01	219.96	290,865	7,203	5,063,251	535,955
New Zealand	18,000	0.40	5,002,100	2,417	26	1.08	483.20	5.20	2,305	86	1,808,406	361,529
El Salvador	16,000	0.30	6,508,877	61,947	1,940	3.13	9,517.31	298.05	58,666	1,341	797,032	122,453
Moldova	8,116	0.20	4,027,448	201,909	4,258	2.11	50,133.24	1,057.25	176,150	21,501	763,992	189,696
Faerol Islands	8,068	16.50	48,992	660	1	0.15	13,471.59	20.41	657	2	234,708	4,790,741
Dominica	7,202	10.00	72,110	156	0	0.00	2,163.36	0.00	141	15	12,325	170,919
Turks and Caicos Islands	6,433	16,6	39,078	2,200	15	0.68	56,297.66	383.85	2,081	104	17,621	450,919
Greenland	5,130	9.00	56,838	31	0	0.00	545.41	0.00	31	0	19,792	348,218
Bilize	4,933	1.20	402,597	12,359	316	2.56	30,698.19	784.90	11,972	71	89,853	223,183
Mongolia	4,926	0.10	3,314,670	3,664	4	0.11	1,105.39	1.21	2,907	753	1,987,062	599,475
Andorra	4,914	6.40	77,351	11,199	112	1.00	144,781.58	1,447.95	10,754	333	193,595	2,502,812
Anguilla	3,929	26.20	15,096	18	0	0.00	1,192.37	0.00	18	0	9,742	645,337
Mauritius	3,843	0.30	1,273,232	711	10	1.41	558.42	7.85	592	109	289,552	227,415

San Marino	3,668	10.80	33,980	4,096	77	1.88	120,541.49	2,266.04	3,550	469	46,902	1,380,283
Liechtenstein	3,126	8.20	38,204	2,601	55	2.11	68,081.88	1,439.64	2,519	27	28,293	740,577
Montenegro	3,062	0.50	628,121	82,610	1,117	1.35	131,519.25	1,778.32	72,490	9,003	303,555	483,275
Greda	3,000	2.70	112,884	148	1	0.68	1,311.08	8.86	147	0	20,288	179,724
Moco	2,400	6.10	39,435	2,097	27	1.29	53,176.11	684.67	1,895	175	51,953	1,317,434
Saint Lucia	2,094	1.10	184,209	3,989	48	1.20	21,654.75	260.57	3,753	188	33,545	182,103
Macao	2,000	0.30	655,442	48	0	0.00	73.23	0.00	41	1	4,369	6,666
Guya	1,852	0.20	789,182	8,993	206	2.29	11,395.34	261.03	8,230	557	73,325	92,913
Falkland Islands	1,732	49.70	3,594	54	0	0.00	15,025.04	0.00	54	0	7,077	1,994,083
Montserrat	652	13.00	4,994	20	1	5.00	4,004.81	200.24	18	1	947	189,628
